# Colistin-resistance genes in *Escherichia coli* isolated from patients with urinary tract infections

**DOI:** 10.1371/journal.pone.0305431

**Published:** 2024-06-12

**Authors:** Waleed M. Al Momani, Nour Ata, Ahmed O. Maslat

**Affiliations:** 1 Department of Basic Medical Sciences, Faculty of Medicine, Yarmouk University, Irbid, Jordan; 2 Department of Biological Sciences, Faculty of Science, Yarmouk University, Irbid, Jordan; Yale University School of Medicine, UNITED STATES

## Abstract

**Background:**

The incidence of antimicrobial resistance is alarmingly high because it occurs in humans, environment, and animal sectors from a “One Health” viewpoint. The emergence of plasmid-carried mobile colistin-resistance (*MCR*) genes limits the efficacy of colistin, which is the last-line treatment for multidrug resistance (MDR) against gram-negative infections.

**Objectives:**

The current study aimed to investigate emergence of colistin-resistance (*MCR* 1–5) genes in *E*. *coli* isolated from patients with urinary tract infections (UTIs) in Jordan.

**Methods:**

*E*. *coli* (n = 132) were collected from urine specimens. The *E*. *coli* isolated from human UTI patients were examined the resistance to colistin based on the presence of *MCR* (1–5). All isolates were tested against 20 antimicrobials using the standard disk diffusion method. The broth microdilution technique was used to analyze colistin resistance. In addition, the *MCR* (1–5) genes were detected using multiplex PCR.

**Results:**

Out of the 132 isolates, 1 isolate was colistin-resistant, having a minimum inhibitory concentration of 8 μg/mL and possessing *MCR*-1. All the *E*. *coli* isolates showed high resistance to penicillin (100%), amoxicillin (79.55%), cephalexin (75.76%), nalidixic acid (62.88%), tetracycline (58.33%), or cefepime (53.79).

**Conclusion:**

To our knowledge, this is the first report on the presence of plasmid-coded *MCR*-1 in *E*. *coli* from a patient with UTIs in Jordan. This is a problematic finding because colistin is the last-line drug for the treatment of infections caused by MDR gram-negative bacteria. There is a crucial need to robustly utilize antibiotics to control and prevent the emergence and prevalence of colistin-resistance genes.

## Introduction

Urinary tract infections (UTIs) caused by antibiotic-resistant gram-negative bacteria (GNB) are the most common bacterial infections encountered by clinicians and are of growing concern owing to limited treatment options [1]. Although other bacteria of the *Enterobacteriaceae* family can cause urinary tract infections (UTIs) [2], *E*. *coli* is the most common etiological agent of UTIs, accounting for up to 80% of all cases [3].

The treatment of UTI is greatly complicated by the emergence of multidrug-resistant (MDR) isolates [4] and the increasing prevalence of MDR *E*. *coli*. The issue of MDR is further exacerbated by the fact that pipeline of innovative antibiotics has been exhausted. In such a scenario, colistin has again come to the limelight in the context of clinical use as the World Health Organization (WHO) has categorized it as one of the antibiotics of crucial significance in human clinical settings [5].

Colistin, the last-line antibiotic to treat acute infections caused by MDR GNB [6], is a narrow-spectrum antibiotic that exhibits robust effects against most members of the *Enterobacteriaceae* family and common non-fermentative GNB [7].

In 2015, researchers in China reported the presence of a plasmid-coded colistin-resistance *MCR*-1 gene in *E*. *coli*. They reported that this gene can be transmitted from one bacterium to another and encodes a phosphoethanolamine transferase that catalyzes the transfer of phosphoethanolamine (a cationic molecule) to lipid A (a key component of LPS), an event that results in altered cell membrane charge, and consequently, the inability of colistin (a cation) to attach to the membrane and induce cell membrane degradation, thereby conferring resistance to colistin [8].

It is thought that *MCR* -1 was derived from animals and then transferred to humans via horizontal transmission. This hypothesis is underscored by the fact that *E*. *coli* isolates harboring *MCR*-1 have been identified in animal food products [9]. The mindless use of colistin in the veterinary sector, particularly in the absence of stringent laws, has contributed to the global spread of *MCR*-1 (10% of animal isolates and 0.1–2% of human isolates), as shown in an Egyptian study [10].

Although studies have reported the presence of *MCR* genes in patients with UTIs in many countries, no study has investigated the prevalence of *MCR* genes in patients with UTIs in Jordan. In the present study, we aimed to elucidate the occurrence of colistin resistance in *E*. *coli* isolates from patients with UTIs in Jordan.

## Materials and methods

### Sample collection and identification

This study was conducted over a period of 6 months (between 15^th^ of June 2022 to 17^th^ and December 2022) and included 132 *E*. *coli* isolates from the urine cultures of patients with UTI. All participants were aged 3–85 years old and all isolates were obtained from Princess Rahma Hospital in Irbid and a clinical diagnostic laboratory in Amman, Jordan. The samples were streaked onto MacConkey, eosin-methylene blue, and blood agar plates (Oxoid, UK). Following incubation at 37°C for 24 h, all isolates were confirmed as *E*. *coli* using standard biochemical tests (IMViC and Kligler Iron Agar tests) and molecular identification tests (polymerase chain reaction (PCR) using the Universal Stress Proteins A (UspA) gene having a band size of 884 bp) [11]. *E*. *coli* NCTC 12900 UK was used as the positive control. This study was approved by the Ethics Committee of Yarmouk University.

### Antimicrobial Susceptibility Testing (AST)

The antibiotic susceptibility profiles of the 132 *E*. *coli* isolates were determined using the disk diffusion technique on Mueller–Hinton agar (Oxoid, UK) using a suspension equivalent in turbidity to 0.5 McFarland. The plates were incubated overnight at 37°C. The results were interpreted according to the guidelines recommended by the Clinical Laboratory Standards Institute (CLSI,2017) [12]. *E*. *coli* isolates were defined as MDR (resistant to three or more antimicrobial classes), based on the International Expert Proposal for Interim Standards Guidelines [13]. Resistance against the following antibiotics was tested: cephalexin (30 μg), penicillin (10 μg), ciprofloxacin (5 μg), doxycycline (30 μg), aztreonam (30 μg), imipenem (10 μg), gentamycin (10 μg), florfenicol (30 μg), kanamycin (30 μg), tigecycline (15 μg), cefepime (30 μg), amoxicillin-clavulanate (30 μg), cefoxitin (30 μg), sulphamethaxazole-trimethoprim (25 μg), chloramphenicol (30 μg), tetracycline (30 μg), fosfomycin (50 μg), meropenem (10 μg), amoxicillin (10 μg), nalidixic acid (30 μg) (Oxoid, UK).

The minimum inhibitory concentration (MIC) of colistin (colistin sulfate powder, DADvet, Jordan) against the 132 *E*. *coli* isolates was determined by microdilution using Muller-Hinton broth (Oxoid, UK). The MIC values ranged from 128 μg/mL to 0.25 μg /mL in a 2-fold dilution order. The clinical breakpoints for colistin resistance were defined according to the European Committee on Antimicrobial Susceptibility Testing (EUCAST) and CLSI statements when the MIC value was >2 μg/mL [14]. *E*. *coli* NCTC 12900 UK was used as a reference strain for disk diffusion and MIC testing.

### Detection of the colistin-resistance genes by multiplex PCR

DNA was extracted using the boiling method [15], briefly, a 300 μL bacterial suspension was prepared from fresh *E*. *coli* colonies grown on nutrient agar (Oxoid, UK), the suspension was vortexed and then incubated in a dry bath (Cleaver, UK) at 100°C for 10 min, followed by immediate incubation on ice for another 10 min. After that, samples were placed in a centrifuge (HERMLE, Germany) and centrifuged at full speed for 10 min, the supernatant was stored at -20°C and used as a template for PCR.

All *E*. *coli* isolates (n = 132) were screened using multiplex PCR to evaluate the presence of mobile colistin-resistance genes *MCR* (1–5). The multiplex PCR assay was performed in accordance with the guidelines proposed by the European Centre for Disease Prevention and Control [16]. The reaction was performed in a total volume of 20 μL (4 μL of 5× HOT FIREPol® Blend Master Mix (Solis BioDyne, Estonia), 4 μL DNA template,6 μL nuclease-free water,1.2 μL of each primer pair) (Table 1). PCR amplification was performed on a Thermocycler (BIO-RAD, USA) with an initial DNA denaturation step at 94°C (15 min), followed by 25 cycles beginning with 30 s of denaturation at 94°C, 90 s of primer annealing at 58°C, and 1 min of extension at 72°C. The final extension step was performed at 72°C for 10 min. Amplified products were visualized by electrophoresis on a 2% agarose gel, followed by staining with ethidium bromide and visualization under UV light.

**Table 1:** PCR target genes of (*Mcr*1-5), primer sequence, PCR product size, and annealing temperature [17].

Target gene	Primer sequence	Product size	
*Mcr*-1	F- 5’AGTCCGTTTGTTCTTGTGGC3’ R- 5’AGATCCTTGGTCTCGGCTTG3’	320 bp	58°C
*Mcr*-2	F- 5’CAAGTGTGTTGGTCGCAGTT3’ R- 5’TCTAGCCCGACAAGCATACC3’	715 bp	58°C
*Mcr*-3	F- 5’AAATAAAAATTGTTCCGCTTATG3’ R-**5’AATGGAGATCCCCGTTTTT3’**	929 bp	58°C
*Mcr*-4	F- 5’TCACTTTCATCACTGCGTTG3’ R- 5’TTGGTCCATGACTACCAATG3’	1116 bp	58°C
*Mcr*-5	F- 5’ATGCGGTTGTCTGCATTTATC3’ R- 5’TCATTGTGGTTGTCCTTTTCTG3’	1644 bp	58°C

### Ethical approval

This study was approved by the Yarmouk Institutional Review Board (IRB No. 2022/32).

## Results and discussion

### Isolation and characterization of *E*. *coli*

**Fig 1:**
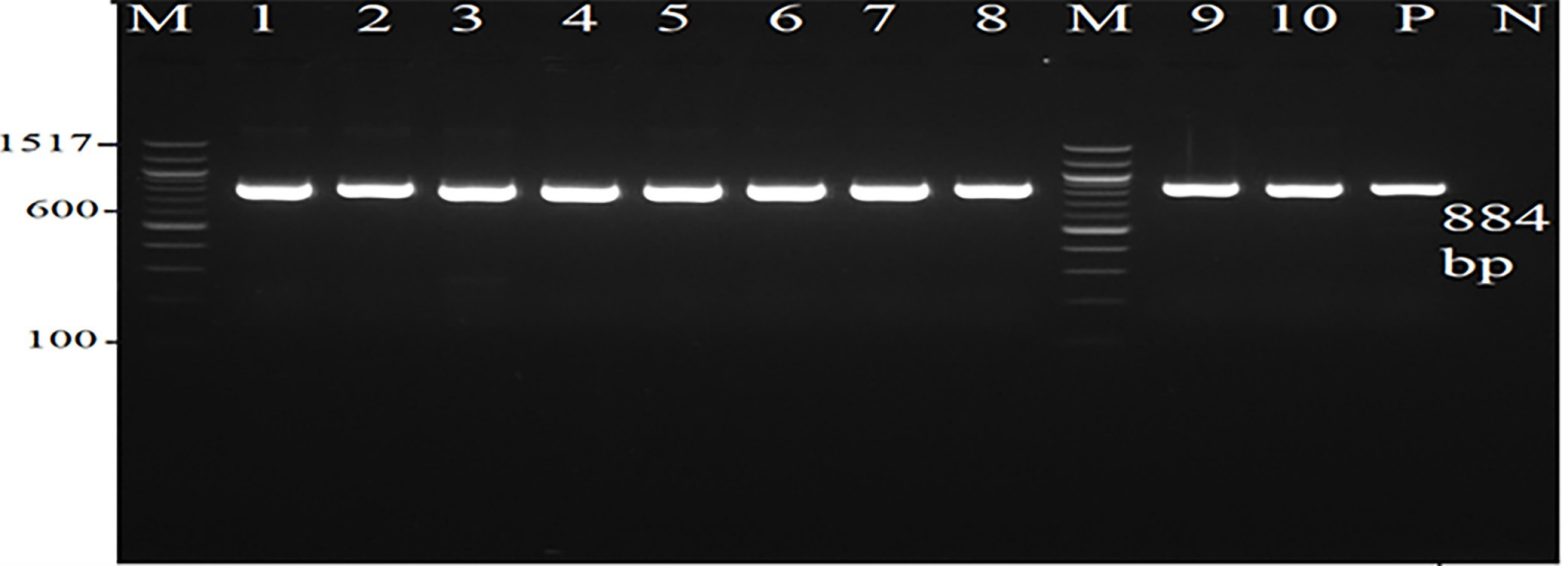
Electrophoresis for *Escherichia coli uspA* gene.

From the 132 urine samples, a total of 132 *E*. *coli* were isolated and confirmed. According to sex, 90.2% (n = 119) of the *E*. *coli* were isolated from females and 9.8% (n = 13) of the *E*. *coli* were isolated from males; according to age, 75% (n = 99) of the *E*. *coli* were isolated from adults, and 25% (n = 33) of the *E*. *coli* were isolated from pediatric individuals. A total of 132 isolates were confirmed as *E*. *coli* by PCR amplification using the Universal Stress Protein A in *Escherichia coli* (UspA gene), some of which are shown in Fig 1.

### Antimicrobial resistance profiles

**Fig 2:**
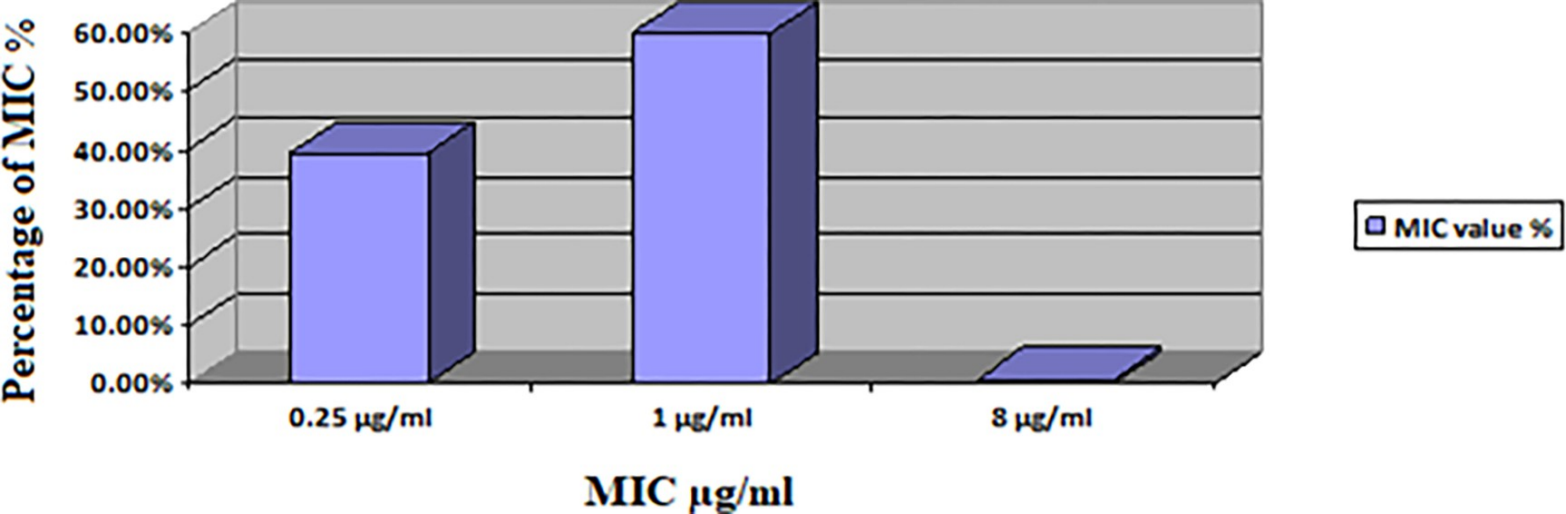
Minimal inhibitory concentration value for *E*. *coli* (n = 132) isolates.

A total of 132 isolates showed high resistance to penicillin (100%), amoxicillin (79.55%), cephalexin (75.76%), nalidixic acid (62.88%), tetracycline (58.33%), and cefepime (53.79%).,. However, resistance was the lowest for fosfomycin (6.06%), florfenicol (10.61%), and chloramphenicol (15.91%). Readings for each antibiotic were recorded in the following three categories: resistant (R), intermediate (I), and susceptible (S). The antibiotic susceptibilities of the isolates for the 20 antimicrobials used in this study are shown in (Table 2 and Fig 2).

**Table 2:** Antibiotic susceptibilities of the 132 *E*. *coli* isolates evaluated using the disk diffusion method.

Class of antibiotics			Antibiotic tested		R	I	S
**1**		**β-lactams**	**1**	**Penicillin**	132(100%)	0(0.0%)	0(0.0%)
			**2**	**Amoxicillin**	105(79.55%)	8(6.1%)	19(14.39%)
			**3**	**Aztreonam**	47(35.61%)	18(13.64%)	67(50.76%)
			**4**	**Imipenem**	7(5.30%)	27(20.45%)	98(74.24%)
			**5**	**Meropenem**	33(25%)	17(13%)	82(62.12%)
**2**		**β-lactamase inhibitors**	**6**	**Amoxicillin-clavulanate**	67(50.76%)	34(25.76%)	31(23.48%)
**3**		**Tetracyclines**	**7**	**Tetracycline (TE)**	77(58.33%)	5(3.79%)	50(37.88%)
			**8**	**Doxycycline (DO)**	50(37.88%)	36(27.27%)	46(34.85%)
			**9**	**Tigecycline (TGC)**	10(7.58%)	23(17.42%)	99(75%)
**4**		**Sulfonamides**	**10**	**Sulphamethaxazole-trimethoprim**	67(50.76%)	7(5.3%)	58(43.94%)
**5**		**Fluoroquinolones**	**11**	**Nalidixic acid**	83(62.88%)	16(12.12%)	33(25%)
			**12**	**Ciprofloxacin**	59(44.70%)	40(30.30%)	33(25%)
**6**		**Aminoglycosides**	**13**	**kanamycin**	28(21.21%)	45(34.1%)	59(44.70%)
			**14**	**Gentamycin**	27(20.45%)	13(9.85%)	92(69.70%)
**7**		**Cephalosporins**	**15**	**Cefepime**	71(53.79%)	15(11.4%)	46(34.85%)
			**16**	**Cefoxitin**	21(15.91%)	14(10.61%)	97(73.48%)
			**17**	**Cephalexin**	100(75.76%)	0 (0.0%)	32 (24.24%)
**8**		**Phosphoric acid derivatives**	**18**	**Fosfomycin**	8(6.06%)	3(2.27%)	121(91.67%)
**9**	**Phenicols**		**19**	**Chloramphenicol**	21(15.91%)	9(6.82%)	102(77.27%)
			**20**	**Florfenicol**	14(10.61%)	1(0.76%)	117(88.64%)

To verify MDR in all the 132 *E*. *coli* isolates, the number of antibiotics each isolate exhibited resistance toward (among a total of 20 antibiotics belonging to different classes) was calculated, and the result has been summarized in (Table 3).

**Table 3:** Summary of the number of *E*. *coli* isolates (n = 132) which are resistant to various antibiotics (n = 20).

Number of antibiotics against which resistance was observed	Number of *E*. *coli* isolates	Percentage %
Resistance to 1 antibiotic	3	2.27%
Resistance to 2 antibiotics	7	5.30%
Resistance to 3 antibiotics	4	3.03%
Resistance to 4 antibiotics	7	5.30%
Resistance to 5 antibiotics	10	7.58%
Resistance to 6 antibiotics	14	10.62%
Resistance to 7 antibiotics	18	13.64%
Resistance to 8 antibiotics	15	11.36%
Resistance to 9 antibiotics	14	10.61%
Resistance to 10 antibiotics	11	8.33%
Resistance to 11 antibiotics	13	9.85%
Resistance to 12 antibiotics	6	4.54%
Resistance to 13 antibiotics	6	4.54%
Resistance to 14 antibiotics	4	3.03%

Each *E*. *coli* isolate was organized according to the number of antibiotic classes against which it showed resistance. Table 4 presents a summary of the resistance profiles of all the 132 *E*. *coli*. Of the 132 *E*. *coli* isolates, 117 exhibited MDR, i.e., 88.64%.

**Table 4:** Summary of the *E*. *coli* isolates (n = 132) which exhibit resistance against various classes of antibiotics to show multidrug resistance.

Number of antibiotic classes against which resistance was observed	Number of *E*. *coli* isolates	Multidrug resistance	Percentage%
Resistance to 1 class	5	No	3.79%
Resistance to 2 classes	10	No	7.57%
Resistance to 3 classes	19	Yes	14.4%
Resistance to 4 classes	16	Yes	12.12%
Resistance to 5 classes	31	Yes	23.5%
Resistance to 6 classes	23	Yes	17.42%
Resistance to 7 classes	21	Yes	15.9%

### Colistin MIC

Among the 132 strains isolated, only a single *E*. *coli* isolate harbored the *MCR*-1 gene and showed resistance to colistin (MIC = 8 μg/mL), The remaining isolates were sensitive to colistin with MIC values < 2 μg/mL (Table 5, Fig 2).

**Table 5:** Minimal Inhibitory Concentration (MIC) values for the 132 *E*. *coli* isolates.

MIC value	MIC = 0.25 μg/mL	MIC = 1 μg/mL	MIC = 8 μg/mL
**Susceptibility**	52(39.39%)	79(59.85%)	-
**Resistance**	-	-	1(0.76)

### Molecular identification of colistin-resistance genes

**Fig 3:**
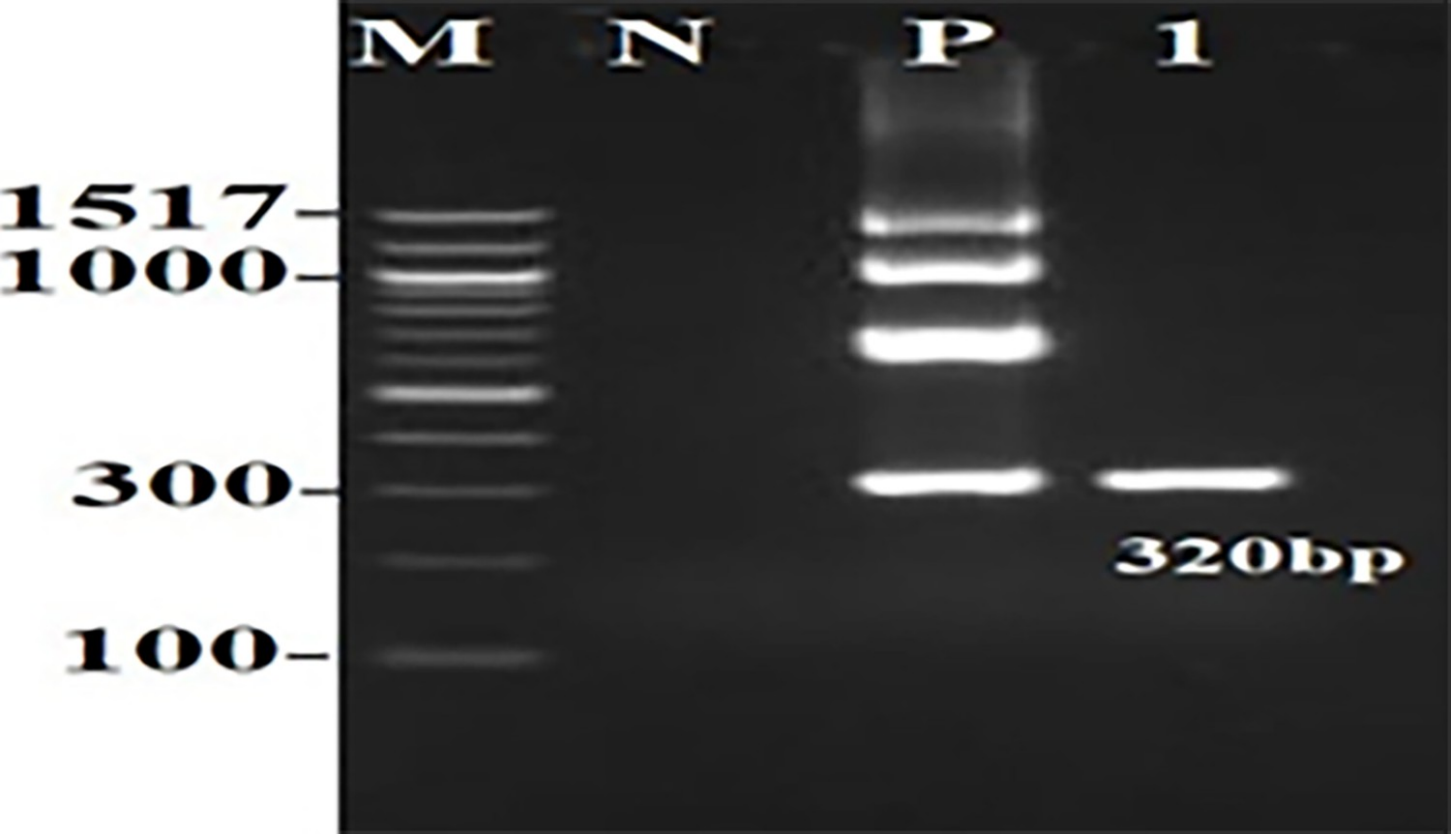
Electrophoresis for the single *E*. *coli* isolate that carried *MCR*-1.

A total of 132 isolates were screened for the presence of *MCR* 1, *MCR* 2, *MCR* 3, *MCR* 4, and *MCR* 5 using multiplex PCR. Our results showed that only 1 of the 132 *E*. *coli* isolates carried *MCR*-1(Fig 3).

### A single *E*. *coli* isolate harbored the *MCR-1* gene

A single *E*. *coli* isolate was deemed resistant to colistin based on the MIC value (8 μg/mL) and the multiplex PCR that detected the presence of *MCR-*1. Summary results for the single *E*. *coli* isolate that showed colistin resistance are presented in Table 6.

**Table 6:** Summary details for the single *E*. *coli* isolate that showed colistin resistance.

Source	Patient Age (years)	No. of isolates	*MCR*	MIC of colistin (μg/mL)	Resistant antibiotics	Number of classes against which resistance is observed
Human UTI	27	1	*MCR*-1	8	AMC, P, CIP, ATM, FEP, CL, SXT, C, TE, NA	7

*E*. *coli* was isolated from urine samples (n = 132), out of which 90.2% (n = 119) originated from females with UTIs while 9.8% (n = 13) originated from males with UTIs, moreover and according to age 25% (n = 33) originated from pediatric individuals while 75% (n = 99) originated from adults. UTIs are one of the major sex-related infectious diseases [18]. Structural differences in the female urinary system contribute to the development of UTIs [19]. An assumption to clarify this difference is that anatomical disparities exist, such as a short space between the anus and urethral opening in females or a long urethra in males [18].

*E*. *coli* is the most common etiological agent of UTI, accounting for up to 80% of all cases [3, 20]. In this study, *MCR*-1 was detected in a single isolate (out of 132, 0.76%) derived from the urine sample of a 27-year-old female patient with a UTI. This prevalence rate was approximately similar to that reported in Myanmar (0.23%) [21], and Switzerland (0.12%) [22]. Despite its low spread, the existence of *MCR*-1 in a clinical isolate in Jordan could be considered a sign that the transmission of resistance genes (mainly *MCR*-1) from *E*. *coli* to humans has been initiated. However, higher prevalence rates have been reported in Egyptian studies with prevalence rates of 22*%*, [23] 5.6*%* [24] and 4.5*%* [25], and another study on UTIs from Bangladesh reported a prevalence rate of 3.52% [26]. The extensive and improper use of antibiotics induces a selective pressure, further complicated by the rapid rise and outbreak of MDR *Enterobacteriaceae* [24].

This study highlights the prevalence of MDR in urinary *E*. *coli*. Finally, 117 of the 132 isolates (88.64%) showed resistance to at least three classes of antibiotics (so as to be called multidrug resistant). This finding is supported by similar studies on UTI showing high rates of resistance to commonly used antibiotics [23]. As a potential justification for such MDR, a high rate of MDR in patients with UTI has been observed as UTI-causing *E*. *coli* are known for their capability to form biofilms that aid recurrence, leading to continuous and resistant infection [27].

Colistin has been used internationally as a last-resort antibiotic for infections caused by GNB [9]. Since its first report in China in 2015, the plasmid-carried colistin-resistance gene *MCR*-1 has been identified in *Enterobacteriaceae* isolated from animals and humans in different countries [28]. The differences in colistin resistance among different studies can be explained by the number of cases, general situation of patients, geographical regions, different antibiotic regulations, and compliance with infection management measures [24]. The mobile colistin-resistant *MCR*-1 is more common than other *MCR* genes, a result that is supported by our study and previous studies [21–25]. In contrast, *MCR*-2 was reported in *E*. *coli* isolated from a patient with a UTI in a study conducted in Bangladesh [26].

All *the E*. *coli* isolates were resistant to penicillin. In addition, highest resistance rates, surpassing 50%, were detected for amoxicillin-clavulanate, cephalexin, cefepime, tetracycline, amoxicillin, nalidixic acid, and sulfamethoxazole-trimethoprim. Moreover, high susceptibility rates, exceeding 75%, were detected for florfenicol, tigecycline, chloramphenicol, and fosfomycin. These resistance rates are similar to those reported in Egypt, demonstrating that *E*. *coli* isolates from patients with UTIs are highly resistant to amoxicillin-clavulanate, nalidixic acid, sulfamethoxazole-trimethoprim, tetracycline, and cefepime [25].

In this study, the highest resistance rates have been found against β-lactams, a possible reason regarding the excessive resistance rate to those antimicrobial agents is that in Jordan—within the previous few years (2012–2015)—ESBL-producing *E*. *coli* (43–54%) have been isolated from UTI patients at a rate that was drastically higher than what was reported in 2009 (10.8%) [29]. The high resistance to these antibiotics may also be due to doctors’ empirical antimicrobial prescription, self-prescription, non-obligation, and drug consumption without permission from the doctor [30].

The detection of *MCR*-1 in an *E*. *coli* isolate from UTI-affected patients was similarly confirmed by multiplex PCR; in a similar manner, *MCR*-1 was detected in colistin-resistant *E*. *coli* isolates from China [31]. A single *E*. *coli* isolate that was resistant to colistin was also resistant to multiple classes of antimicrobials but was susceptible to gentamicin, florfenicol, kanamycin, tigecycline, and fosfomycin. Several studies have shown that colistin-resistant isolates are highly resistant to multiple classes of antimicrobials [24].

In the current study, the isolate was deemed resistant to colistin based on the MIC. This *E*. *coli* isolate carrying the *MCR*-1 gene exhibited an MIC value of 8 μg/mL (MIC > 2 μg/mL). These results are similar to those of studies performed in Egypt and Saudi Arabia [25]. Among the 132 strains isolated, a single *E*. *coli* isolate harbored the *MCR-*1 gene and exhibited resistance to colistin (MIC = 8 μg/mL). The remaining isolates were sensitive to colistin with MIC values < 2 μg/mL. These results are similar to studies in Egypt [23, 25]. However, other studies have shown that some isolates that come out negative for the presence of resistance genes in PCR exhibit phenotypic colistin resistance at the MIC [24, 26].

The MIC values for resistant isolates ranged from 2 to 128 μg/mL, and 23 (6.4%) isolates exhibited MIC values of ≥ 8 μg/mL in Jordanian study [32]. In addition, *E*. *coli* isolates from broilers have been reported to display the highest resistance to tetracycline 360 (100%), penicillin 359 (99.7%), and amoxicillin 357 (99.2%) [32]. The worldwide increase in the spread of *MCR*-1 among animal isolates (compared to human clinical isolates) implies that animals are the probable sources of *MCR*-1 present in humans. Furthermore, misuse of colistin in agriculture and poultry may be the principal reason for the elevated prevalence of *MCR*-1 in bacteria isolated from animals and animal yields [32, 33]. In veterinary science, colistin is used for various purposes, including the prophylaxis and treatment of enteric infections, in addition to its use as a dietary supplement in poultry farms to prevent infections caused by pathogenic bacteria [23].

This study provides data on the antimicrobial resistance patterns of *E*. *coli* isolated from patients with UTIs. We concentrated on UTIs because they still represent a major source of infection in humans [22]. *E*. *coli* from community-received infections involve the interplay between the environment and hospitals, playing a possible role as an exchange platform for *MCR*-like genes within the environment [22], and *E*. *coli* is the most common member of *Enterobacteriaceae* isolated from clinical samples [24].

The current study had several fundamental limitations, such as the cross-sectional design without future follow-up due to resource limitations, and the fact that recurrent infections were not sequestered from first-time infections. The data in the current study show a worrisome spread of *MCR*-1-carrying colistin-resistant *E*. *coli*, as found in previous studies in Jordan in humans and broilers [32].

These results may reflect the prevalence of colistin-resistant *E*. *coli* in Jordan or the silent spread of *MCR*-1 in humans. Furthermore, analysis of the genetic data of *MCR*-1-positive strains could help us understand the origin of this gene. The presence of *MCR* in this study indicates a massive public health issue; this is especially important as colistin antibiotics are the last-line drugs for infection treatment. *MCR* genes are carried by plasmids and can be spread via horizontal gene transfer to other commensal and pathogenic bacteria [34, 35]. A coordinated strategy for determining *MCR*-1 dissemination is needed to restrict the spread of multidrug-resistant isolates among patients [24]. More stringent rules must be enforced to halt the further dissemination of colistin-resistant genes [26].

## Conclusions

Colistin is considered one of the last lines of therapy that had been used to treat extreme infections caused by MDR pathogens. The development of plasmid-mediated colistin resistance in *E*. *coli* poses a serious problem due to its high potential for spreading in medical settings. *MCR*-1 has been reported in most continents and has been observed in numerous bacterial isolates, especially *E*. *coli*, from animals, humans, and the environment. In Jordan, colistin-resistant *E*. *coli* harboring *MCR*-1 was recorded for the first time in patients with UTIs. This highlights the potential health risks that plasmid-carried colistin-resistant genes in *E*. *coli* can be detrimental to millions of humans in Jordan. In addition, guidelines should be established on the use of colistin in the human and animal sectors to ensure the success of the therapy and prevent the spread of these resistance genes.

## Supporting information

S1 Raw data (PDF)

S1 Raw images (PDF)
