# Supplementary material for: Colistin-resistance genes in Escherichia coli isolated from patients with urinary tract infections
Source: PLoS One. 2024 Jun 12;19(6):e0305431. doi: 10.1371/journal.pone.0305431 (PMC11168671; doi:10.1371/journal.pone.0305431)
Supplement: S1 Raw data — (PDF) [file pone.0305431.s001.pdf]

Penicillin			Amoxicillin			Aztreonam	
Interpretation	Mean	SD	Interpretation	Mean	SD	Interpretation	Mean
R	0	0	R	20.00	1.00	R	16.67
R	0	0	R	19.67	2.08	R	16.00
R	0	0	R	19.00	1.73	R	16.33
R	0	0	R	20.00	1.73	R	15.67
R	0	0	R	19.67	2.52	R	15.67
R	0	0	R	20.67	1.53	R	16.67
R	0	0	R	19.00	1.00	R	16.67
R	0	0	R	18.33	0.58	R	16.67
R	0	0	R	19.33	2.08	R	15.67
R	0	0	R	18.67	2.08	R	15.67
R	0	0	R	20.33	0.58	R	15.33
R	0	0	R	19.67	1.53	R	16.00
R	0	0	R	19.33	1.15	R	15.33
R	0	0	R	18.67	2.08	R	16.00
R	0	0	R	22.00	0.00	R	16.00
R	0	0	R	19.33	2.31	R	16.67
R	0	0	R	18.33	0.58	R	17.00
R	0	0	R	18.67	1.15	R	16.00
R	0	0	R	19.33	2.52	R	15.33
R	0	0	R	19.33	2.52	R	16.33
R	0	0	R	19.33	2.08	R	16.00
R	0	0	R	20.33	2.08	R	16.00
R	0	0	R	20.00	1.73	R	16.00
R	0	0	R	18.00	1.00	R	16.00
R	0	0	R	20.00	1.73	R	16.00
R	0	0	R	19.67	2.52	R	15.33
R	0	0	R	18.33	1.53	R	17.00
R	0	0	R	19.00	1.00	R	15.33
R	0	0	R	20.33	1.15	R	15.67
R	0	0	R	19.33	0.58	R	16.33
R	0	0	R	21.00	1.73	R	15.33
R	0	0	R	19.33	1.53	R	15.67
R	0	0	R	18.67	1.53	R	15.67
R	0	0	R	20.00	2.00	R	16.67
R	0	0	R	18.00	1.00	R	15.00
R	0	0	R	20.67	2.31	R	15.33
R	0	0	R	20.33	2.08	R	15.67
R	0	0	R	19.67	0.58	R	16.33
R	0	0	R	19.00	2.00	R	16.67
R	0	0	R	18.67	2.08	R	16.33
R	0	0	R	19.00	1.00	R	15.33
R	0	0	R	19.00	1.73	R	16.67
R	0	0	R	21.00	1.73	R	15.00
R	0	0	R	20.00	1.73	R	17.00
R	0	0	R	18.33	0.58	R	16.67

R	0	0	R	20.00	2.00	R	16.00
R	0	0	R	20.33	1.15	R	16.00
R	0	0	R	21.00	1.00	I	20.00
R	0	0	R	19.00	2.65	I	20.33
R	0	0	R	18.33	0.58	I	19.67
R	0	0	R	19.33	2.31	I	20.33
R	0	0	R	19.67	1.53	I	19.33
R	0	0	R	21.33	1.15	I	20.67
R	0	0	R	19.33	0.58	I	19.33
R	0	0	R	20.33	1.53	I	20.33
R	0	0	R	20.33	2.89	I	20.00
R	0	0	R	21.00	1.00	I	20.00
R	0	0	R	19.33	1.53	I	20.00
R	0	0	R	19.33	2.08	I	20.00
R	0	0	R	20.00	1.73	I	20.00
R	0	0	R	18.33	1.15	I	20.33
R	0	0	R	19.67	2.31	I	20.00
R	0	0	R	20.33	0.58	I	19.67
R	0	0	R	19.00	2.65	I	20.33
R	0	0	R	19.33	1.53	I	19.00
R	0	0	R	21.33	1.15	S	22.00
R	0	0	R	21.00	1.00	S	23.33
R	0	0	R	19.00	2.65	S	23.67
R	0	0	R	19.67	1.53	S	23.33
R	0	0	R	20.67	1.53	S	24.33
R	0	0	R	18.00	1.00	S	22.67
R	0	0	R	19.33	1.15	S	22.67
R	0	0	R	20.33	1.53	S	24.00
R	0	0	R	18.67	2.08	S	23.33
R	0	0	R	20.00	2.65	S	22.00
R	0	0	R	18.00	1.73	S	23.67
R	0	0	R	19.67	2.31	S	23.67
R	0	0	R	18.67	2.08	S	23.00
R	0	0	R	20.00	0.00	S	24.33
R	0	0	R	20.33	0.58	S	22.33
R	0	0	R	20.67	1.53	S	23.33
R	0	0	R	20.00	2.00	S	23.33
R	0	0	R	19.67	2.52	S	23.67
R	0	0	R	20.00	1.73	S	24.00
R	0	0	R	19.67	1.53	S	23.67
R	0	0	R	20.33	2.89	S	23.00
R	0	0	R	20.00	2.00	S	23.00
R	0	0	R	19.67	2.31	S	24.00
R	0	0	R	18.33	1.53	S	24.00
R	0	0	R	19.00	1.00	S	23.00
R	0	0	R	20.33	2.89	S	22.67
R	0	0	R	19.33	0.58	S	22.67

R	0	0	R	18.33	0.58	S	22.67
R	0	0	R	17.67	0.58	S	23.67
R	0	0	R	18.33	1.15	S	24.00
R	0	0	R	20.00	2.00	S	23.33
R	0	0	R	18.00	1.00	S	24.33
R	0	0	R	19.33	2.52	S	22.33
R	0	0	R	20.00	2.00	S	24.67
R	0	0	R	18.67	2.89	S	24.33
R	0	0	R	20.67	1.53	S	23.33
R	0	0	R	18.67	1.53	S	23.67
R	0	0	R	20.00	2.65	S	23.67
R	0	0	R	20.33	2.89	S	24.33
R	0	0	R	19.67	2.31	S	23.67
R	0	0	I	24.00	1.00	S	23.67
R	0	0	I	23.67	0.58	S	23.33
R	0	0	I	24.33	1.15	S	23.33
R	0	0	I	23.67	1.15	S	23.33
R	0	0	I	24.00	1.00	S	23.33
R	0	0	I	23.67	0.58	S	24.33
R	0	0	I	24.33	1.15	S	24.33
R	0	0	I	23.67	1.15	S	24.33
R	0	0	S	29.67	0.58	S	23.67
R	0	0	S	29.00	1.73	S	23.00
R	0	0	S	28.00	1.00	S	23.67
R	0	0	S	28.67	1.15	S	23.33
R	0	0	S	29.67	0.58	S	24.67
R	0	0	S	28.67	0.58	S	24.00
R	0	0	S	28.67	0.58	S	24.00
R	0	0	S	29.33	0.58	S	23.00
R	0	0	S	28.00	1.00	S	23.67
R	0	0	S	27.67	1.15	S	23.33
R	0	0	S	29.33	1.15	S	23.33
R	0	0	S	27.67	0.58	S	24.00
R	0	0	S	28.00	1.00	S	24.33
R	0	0	S	28.67	0.58	S	23.33
R	0	0	S	28.33	1.53	S	23.67
R	0	0	S	29.00	1.73	S	24.33
R	0	0	S	28.33	1.53	S	24.67
R	0	0	S	28.67	1.53	S	24.33
R	0	0	S	29.00	0.00	S	24.00

	Imipenem			Meropenem			Amoxicil
SD	Interpretation	Mean	SD	Interpretation	Mean	SD	Interpretation
0.58	R	17.00	1.00	R	17.00	2.00	R
1.00	R	16.67	2.08	R	16.67	1.53	R
1.15	R	16.33	2.31	R	17.33	1.53	R
0.58	R	16.33	2.31	R	17.33	1.15	R
1.15	R	17.33	1.53	R	17.33	2.08	R
0.58	R	16.67	1.15	R	16.33	1.53	R
0.58	R	17.33	2.08	R	17.33	2.08	R
0.58	I	21.00	1.00	R	17.00	1.73	R
1.15	I	21.33	1.15	R	16.33	2.31	R
1.15	I	22.67	0.58	R	18.33	1.15	R
0.58	I	21.67	1.15	R	17.67	2.31	R
1.00	I	22.33	0.58	R	17.33	1.15	R
0.58	I	21.00	1.73	R	15.33	0.58	R
1.00	I	22.33	0.58	R	16.67	0.58	R
1.00	I	20.67	0.58	R	17.67	1.53	R
0.58	I	22.33	0.58	R	17.67	2.31	R
0.00	I	21.67	1.53	R	16.33	1.53	R
1.00	I	21.33	1.53	R	16.00	1.00	R
0.58	I	20.67	0.58	R	17.67	0.58	R
0.58	I	21.67	1.53	R	15.67	1.15	R
1.00	I	21.33	0.58	R	17.00	2.00	R
1.00	I	20.00	0.00	R	18.33	0.58	R
1.00	I	21.33	1.53	R	18.00	1.00	R
1.00	I	21.00	1.73	R	18.00	1.73	R
1.00	I	21.67	0.58	R	18.67	0.58	R
0.58	I	22.00	1.73	R	17.00	2.00	R
0.00	I	22.67	0.58	R	16.67	1.15	R
0.58	I	22.67	0.58	R	15.67	0.58	R
0.58	I	22.00	1.00	R	18.33	0.58	R
1.15	I	21.00	1.00	R	17.67	2.31	R
0.58	I	20.67	0.58	R	16.67	1.53	R
0.58	I	20.67	0.58	R	16.67	1.53	R
1.15	I	22.00	1.73	R	17.67	2.31	R
0.58	I	22.67	0.58	I	20.67	0.58	R
0.00	S	24.67	1.15	I	20.33	0.58	R
0.58	S	25.33	1.15	I	20.33	0.58	R
0.58	S	26.00	0.00	I	22.00	1.73	R
0.58	S	25.00	1.00	I	20.67	1.15	R
0.58	S	25.67	0.58	I	20.33	0.58	R
0.58	S	24.67	0.58	I	21.67	1.15	R
0.58	S	26.00	0.00	I	23.00	0.00	R
0.58	S	25.00	1.00	I	22.67	0.58	R
0.00	S	24.67	0.58	I	21.33	0.58	R
0.00	S	25.33	1.15	I	21.33	1.53	R
0.58	S	25.33	0.58	I	22.33	1.15	R

1.00	S	25.00	1.00	I	21.67	0.58	R
1.00	S	25.33	0.58	I	21.00	1.00	R
1.00	S	25.33	0.58	I	21.00	1.73	R
0.58	S	24.33	0.58	I	22.00	1.00	R
1.15	S	25.00	1.00	I	21.67	1.15	R
0.58	S	25.00	0.00	S	25.33	0.58	R
0.58	S	24.33	0.58	S	24.67	0.58	R
0.58	S	24.67	0.58	S	24.67	1.15	R
0.58	S	24.67	0.58	S	25.00	1.00	R
1.15	S	24.33	0.58	S	25.33	1.15	R
0.00	S	25.00	0.00	S	24.00	0.00	R
1.00	S	24.67	0.58	S	24.67	0.58	R
1.00	S	25.33	0.58	S	24.67	0.58	R
1.00	S	25.67	0.58	S	25.00	1.00	R
1.00	S	24.33	0.58	S	25.33	1.15	R
1.15	S	24.67	0.58	S	25.67	0.58	R
1.00	S	24.33	0.58	S	25.67	0.58	R
1.15	S	25.33	0.58	S	24.67	1.15	R
1.15	S	25.67	0.58	S	24.67	1.15	R
0.00	S	25.00	1.00	S	24.67	1.15	R
0.00	S	25.00	1.00	S	25.00	0.00	R
1.53	S	26.00	0.00	S	24.67	1.15	R
1.53	S	25.00	1.00	S	25.00	1.00	I
1.15	S	25.00	0.00	S	25.00	1.00	I
0.58	S	25.67	0.58	S	25.00	1.00	I
1.15	S	25.00	1.00	S	24.33	0.58	I
1.15	S	24.67	1.15	S	25.67	0.58	I
1.73	S	25.67	0.58	S	25.00	1.00	I
1.53	S	24.67	0.58	S	24.00	0.00	I
0.00	S	25.00	1.00	S	25.33	0.58	I
1.53	S	25.00	1.00	S	24.33	0.58	I
1.53	S	25.33	0.58	S	24.67	1.15	I
1.73	S	25.00	1.00	S	25.00	1.00	I
1.15	S	25.00	1.00	S	24.67	0.58	I
0.58	S	24.67	1.15	S	25.67	0.58	I
1.53	S	24.67	1.15	S	24.33	0.58	I
1.53	S	25.00	1.00	S	25.33	0.58	I
1.53	S	25.00	1.00	S	24.67	1.15	I
1.00	S	25.00	1.00	S	24.33	0.58	I
1.15	S	24.67	0.58	S	25.67	0.58	I
1.00	S	25.00	0.00	S	24.33	0.58	I
0.00	S	24.00	0.00	S	25.33	1.15	I
1.00	S	24.00	0.00	S	24.67	1.15	I
1.00	S	25.67	0.58	S	25.33	0.58	I
1.00	S	24.33	0.58	S	25.67	0.58	I
1.15	S	25.33	1.15	S	25.00	1.00	I
0.58	S	24.67	1.15	S	25.33	0.58	I

1.15	S	25.67	0.58	S	25.33	1.15	I
1.53	S	25.00	1.00	S	25.00	0.00	I
1.00	S	25.00	1.00	S	25.33	1.15	I
1.53	S	25.33	1.15	S	24.67	0.58	I
0.58	S	26.00	0.00	S	25.67	0.58	I
0.58	S	25.33	0.58	S	25.67	0.58	I
0.58	S	26.00	0.00	S	25.00	1.00	I
0.58	S	25.00	0.00	S	24.67	1.15	I
1.53	S	25.33	1.15	S	25.00	1.00	I
0.58	S	24.00	0.00	S	25.33	0.58	S
1.53	S	25.33	0.58	S	26.00	0.00	S
0.58	S	24.67	1.15	S	25.33	1.15	S
1.53	S	25.00	1.00	S	25.00	1.00	S
0.58	S	26.00	0.00	S	25.00	1.00	S
1.53	S	24.67	0.58	S	25.67	0.58	S
1.53	S	25.67	0.58	S	25.00	1.00	S
0.58	S	25.33	1.15	S	25.00	1.00	S
1.53	S	24.33	0.58	S	25.00	1.00	S
0.58	S	25.67	0.58	S	24.67	1.15	S
0.58	S	24.00	0.00	S	25.00	1.00	S
0.58	S	25.00	1.00	S	24.67	0.58	S
1.53	S	24.00	0.00	S	24.67	0.58	S
1.00	S	26.00	0.00	S	25.33	1.15	S
1.53	S	25.67	0.58	S	25.33	1.15	S
1.53	S	25.00	1.00	S	24.33	0.58	S
0.58	S	25.33	0.58	S	24.33	0.58	S
1.00	S	25.67	0.58	S	24.33	0.58	S
1.73	S	25.33	0.58	S	24.67	1.15	S
1.00	S	25.33	0.58	S	25.00	0.00	S
1.53	S	24.67	0.58	S	25.67	0.58	S
1.53	S	24.33	0.58	S	24.67	0.58	S
1.53	S	25.33	0.58	S	24.33	0.58	S
1.73	S	25.67	0.58	S	25.33	1.15	S
0.58	S	25.00	1.00	S	24.67	1.15	S
1.15	S	25.33	0.58	S	24.00	0.00	S
0.58	S	25.00	1.00	S	26.00	0.00	S
1.15	S	25.00	1.00	S	26.00	0.00	S
0.58	S	25.00	1.00	S	24.67	0.58	S
1.15	S	25.67	0.58	S	24.33	0.58	S
1.73	S	24.67	1.15	S	25.33	0.58	S

llin-clavulanate		Tetracycline			Doxycycline		
Mean	SD	Interpretation	Mean	SD	Interpretation	Mean	SD
12.67	0.58	R	10.00	1.00	R	9.67	0.58
12.33	0.58	R	9.67	1.15	R	9.33	0.58
11.00	1.00	R	10.33	1.15	R	8.00	0.00
11.00	0.00	R	10.67	0.58	R	8.33	0.58
11.67	1.53	R	9.67	1.15	R	8.33	0.58
11.33	1.53	R	10.33	1.15	R	8.33	0.58
11.33	1.15	R	9.67	0.58	R	8.00	0.00
12.00	1.00	R	10.67	0.58	R	8.67	0.58
11.67	1.53	R	9.67	1.15	R	9.33	0.58
11.67	1.15	R	10.67	0.58	R	8.67	1.15
12.00	1.00	R	9.67	1.15	R	9.00	1.00
11.67	1.53	R	9.67	0.58	R	8.67	0.58
11.67	1.15	R	10.00	1.00	R	9.33	0.58
11.33	1.53	R	10.00	1.00	R	8.00	0.00
10.33	0.58	R	10.00	1.00	R	8.67	1.15
11.00	1.73	R	10.67	0.58	R	9.33	0.58
11.33	0.58	R	9.67	0.58	R	9.00	1.00
11.33	1.53	R	9.67	1.15	R	9.00	1.00
12.67	0.58	R	10.33	0.58	R	8.33	0.58
11.00	1.73	R	10.33	1.15	R	8.67	1.15
11.00	1.73	R	10.33	1.15	R	9.33	1.15
12.00	1.73	R	10.00	1.00	R	8.33	0.58
10.67	1.15	R	10.00	1.00	R	8.67	0.58
11.00	1.00	R	10.33	1.15	R	9.67	0.58
12.33	0.58	R	10.33	1.15	R	8.67	1.15
11.33	1.53	R	10.00	1.00	R	8.33	0.58
11.67	0.58	R	9.67	1.15	R	8.33	0.58
12.00	1.00	R	10.00	1.00	R	9.00	0.00
12.67	0.58	R	10.33	1.15	R	8.67	0.58
11.00	0.00	R	9.33	0.58	R	9.67	0.58
10.33	0.58	R	10.67	0.58	R	8.67	1.15
11.33	0.58	R	11.00	0.00	R	9.00	1.00
12.00	1.73	R	10.00	1.00	R	9.00	1.00
11.00	1.00	R	10.33	0.58	R	8.67	0.58
12.67	0.58	R	10.33	0.58	R	9.67	0.58
11.67	1.15	R	9.33	0.58	R	9.33	0.58
11.00	1.00	R	9.67	0.58	R	10.00	0.00
10.67	1.15	R	10.00	1.00	R	9.33	0.58
10.67	1.15	R	10.33	0.58	R	9.00	1.00
11.33	1.53	R	10.00	1.00	R	8.00	0.00
12.33	0.58	R	10.33	0.58	R	8.33	0.58
12.00	0.00	R	10.00	1.00	R	9.00	1.00
11.67	1.53	R	10.67	0.58	R	9.00	1.00
10.67	1.15	R	9.67	1.15	R	9.00	1.00
10.67	0.58	R	10.00	0.00	R	8.33	0.58

12.00	1.00	R	9.33	0.58	R	8.67	1.15
10.67	1.15	R	10.33	1.15	R	8.00	0.00
11.67	1.15	R	9.67	1.15	R	9.00	1.00
11.67	1.53	R	9.00	0.00	R	9.00	1.00
13.00	0.00	R	10.00	1.00	R	8.67	0.58
11.67	1.53	R	10.33	0.58	I	12.00	1.00
11.33	1.53	R	10.00	1.00	I	12.00	1.00
12.00	1.73	R	9.33	0.58	I	12.33	0.58
12.67	0.58	R	9.67	0.58	I	11.67	0.58
10.67	1.15	R	10.33	0.58	I	11.67	0.58
11.67	0.58	R	10.00	1.00	I	12.33	0.58
11.33	0.58	R	9.67	0.58	I	11.67	1.15
11.33	1.53	R	9.67	0.58	I	12.00	1.00
12.67	0.58	R	10.33	0.58	I	12.33	0.58
12.00	0.00	R	9.33	0.58	I	11.67	1.15
12.00	0.00	R	10.00	1.00	I	12.67	0.58
11.67	1.53	R	10.00	1.00	I	11.67	0.58
11.33	1.53	R	10.00	1.00	I	11.67	0.58
11.00	1.00	R	9.67	1.15	I	11.67	0.58
10.33	0.58	R	10.00	1.00	I	11.67	1.15
12.00	1.00	R	10.33	1.15	I	11.33	0.58
11.67	1.15	R	9.33	0.58	I	12.67	0.58
16.00	1.00	R	9.67	1.15	I	11.33	0.58
16.33	1.15	R	10.67	0.58	I	12.00	1.00
15.67	0.58	R	9.33	0.58	I	12.33	1.15
16.67	0.58	I	12.33	0.58	I	11.00	0.00
17.00	0.00	I	13.33	0.58	I	11.33	0.58
16.33	1.15	I	14.67	0.58	I	12.00	1.00
16.33	0.58	I	12.33	0.58	I	12.00	1.00
16.00	1.00	I	13.00	1.00	I	11.67	0.58
15.67	0.58	S	18.00	2.00	I	11.67	0.58
15.67	1.15	S	19.00	1.00	I	12.33	0.58
16.00	0.00	S	17.67	0.58	I	12.00	1.00
16.00	1.00	S	18.00	1.00	I	12.33	1.15
16.33	1.15	S	17.00	1.73	I	12.67	0.58
15.67	1.15	S	17.00	1.73	I	12.33	0.58
16.33	0.58	S	18.00	2.00	I	11.33	0.58
16.33	1.15	S	19.00	1.00	I	12.00	0.00
16.00	1.00	S	19.00	1.00	I	12.33	1.15
16.00	1.00	S	18.67	2.31	I	11.67	0.58
16.67	0.58	S	18.00	1.00	I	12.00	1.00
15.67	0.58	S	17.67	2.08	S	16.33	2.08
16.33	0.58	S	18.33	0.58	S	16.00	1.00
15.67	1.15	S	18.33	1.53	S	16.00	1.00
16.00	1.00	S	19.00	1.73	S	17.67	0.58
15.67	0.58	S	19.00	0.00	S	16.67	1.15
16.33	0.58	S	17.00	1.00	S	15.67	2.08

16.00	1.00	S	18.00	1.73	S	15.00	1.00
16.00	1.00	S	18.33	1.15	S	16.00	1.73
16.00	0.00	S	17.67	2.08	S	15.33	0.58
16.33	0.58	S	18.00	2.00	S	16.00	1.73
17.00	0.00	S	19.67	0.58	S	15.67	2.08
15.67	1.15	S	19.33	0.58	S	16.67	1.15
16.00	1.00	S	17.00	1.73	S	15.00	1.00
16.00	1.00	S	17.67	0.58	S	17.67	0.58
19.33	0.58	S	18.00	1.73	S	15.67	2.08
21.33	0.58	S	16.67	1.15	S	17.33	0.58
19.67	0.58	S	18.00	2.00	S	15.00	1.73
20.67	0.58	S	16.67	1.15	S	14.00	0.00
20.67	1.53	S	16.67	0.58	S	16.00	1.73
20.33	1.15	S	18.33	1.53	S	14.67	0.58
19.00	0.00	S	18.67	1.53	S	15.67	1.15
20.00	1.73	S	17.33	2.31	S	15.67	0.58
21.33	1.15	S	19.00	1.73	S	15.67	1.15
21.00	1.00	S	17.67	1.53	S	16.67	1.53
21.33	1.15	S	18.33	1.53	S	16.33	2.08
20.00	1.73	S	18.33	2.08	S	16.33	2.08
19.67	1.15	S	19.00	1.73	S	16.00	1.73
20.67	1.53	S	18.67	0.58	S	16.00	2.00
19.33	0.58	S	18.33	1.53	S	17.33	1.15
19.67	1.15	S	16.67	1.15	S	15.33	2.31
20.33	1.15	S	17.67	1.53	S	15.00	1.00
20.67	1.53	S	17.00	1.73	S	16.67	0.58
20.00	1.00	S	19.67	0.58	S	16.67	1.53
21.00	1.00	S	16.67	0.58	S	16.00	1.73
19.67	0.58	S	17.33	1.53	S	16.67	2.31
20.33	1.53	S	17.67	1.15	S	16.00	1.00
21.00	1.00	S	17.33	1.53	S	16.00	2.00
20.00	1.73	S	17.33	1.53	S	17.00	1.73
19.67	0.58	S	19.33	0.58	S	15.67	2.08
21.00	1.73	S	18.67	2.31	S	17.00	1.00
20.33	1.15	S	17.67	1.15	S	16.33	1.53
21.00	1.00	S	18.00	1.00	S	15.33	1.15
19.67	1.15	S	17.67	2.08	S	15.33	2.31
20.00	1.73	S	18.33	2.08	S	16.67	2.31
21.00	1.00	S	16.00	0.00	S	17.00	1.00
19.67	1.15	S	18.00	1.00	S	16.33	1.15

Tigecycline			Sulphamethaxazole-trimethoprim			Nalidixic-acid	
Interpretation	Mean	SD	Interpretation	Mean	SD	Interpretation	Mean
R	14.67	0.58	R	8.67	1.15	R	12.67
R	14.00	1.00	R	8.33	1.53	R	11.33
R	13.33	0.58	R	7.33	0.58	R	13.00
R	14.00	1.00	R	7.67	0.58	R	10.67
R	14.33	1.15	R	7.33	0.58	R	10.67
R	14.67	0.58	R	9.00	1.00	R	11.67
R	14.00	1.00	R	8.33	1.53	R	11.00
R	14.33	0.58	R	9.33	0.58	R	11.67
R	13.33	0.58	R	8.00	1.00	R	10.67
R	14.67	0.58	R	9.00	0.00	R	11.33
I	16.67	1.15	R	8.00	1.73	R	12.00
I	16.00	0.00	R	9.33	1.15	R	11.67
I	16.33	0.58	R	9.00	1.00	R	12.00
I	17.33	1.15	R	9.00	1.00	R	11.67
I	17.67	0.58	R	7.33	0.58	R	11.67
I	16.67	1.15	R	9.67	0.58	R	11.67
I	16.67	1.15	R	8.00	1.00	R	11.33
I	17.00	1.00	R	9.00	1.73	R	12.33
I	17.00	1.00	R	9.33	1.15	R	11.00
I	17.00	1.00	R	8.67	1.15	R	11.33
I	16.67	0.58	R	7.67	0.58	R	12.33
I	17.33	0.58	R	9.00	1.73	R	11.33
I	16.33	0.58	R	9.00	1.73	R	11.00
I	17.33	1.15	R	8.00	1.00	R	11.67
I	17.00	1.00	R	9.33	1.15	R	11.00
I	17.00	1.00	R	9.00	1.00	R	12.00
I	16.33	0.58	R	9.00	1.00	R	11.67
I	16.67	1.15	R	8.00	1.00	R	11.67
I	16.67	0.58	R	9.67	0.58	R	11.00
I	18.00	0.00	R	7.33	0.58	R	12.00
I	17.67	0.58	R	9.00	1.00	R	10.67
I	17.67	0.58	R	9.33	1.15	R	12.00
I	16.33	0.58	R	8.00	0.00	R	11.67
S	21.67	0.58	R	7.67	0.58	R	10.67
S	20.33	1.53	R	9.67	0.58	R	12.33
S	20.67	1.53	R	8.33	0.58	R	10.00
S	21.00	1.00	R	9.33	0.58	R	12.33
S	21.33	1.15	R	7.67	0.58	R	10.33
S	21.00	1.00	R	8.00	1.00	R	11.33
S	19.67	1.15	R	9.00	1.73	R	12.00
S	21.00	1.73	R	8.00	1.73	R	11.00
S	21.00	1.00	R	8.67	1.53	R	11.67
S	22.00	0.00	R	9.00	1.00	R	12.67
S	21.33	1.15	R	9.00	1.73	R	10.67
S	20.33	0.58	R	8.33	1.53	R	12.00

S	20.33	1.15	R	9.33	0.58	R	11.33
S	21.00	1.00	R	8.00	1.00	R	11.67
S	21.00	1.00	R	8.00	0.00	R	11.00
S	20.00	1.00	R	8.33	1.15	R	12.00
S	21.00	1.73	R	7.33	0.58	R	11.33
S	21.00	1.73	R	9.33	1.15	R	11.00
S	21.67	0.58	R	8.33	1.53	R	12.33
S	19.33	0.58	R	9.33	0.58	R	11.67
S	21.00	1.73	R	9.00	1.73	R	10.67
S	20.67	1.53	R	9.33	0.58	R	11.67
S	20.67	1.53	R	8.33	1.53	R	12.00
S	20.00	1.73	R	7.67	0.58	R	11.67
S	20.33	0.58	R	8.00	1.73	R	11.67
S	20.00	1.00	R	8.33	1.15	R	12.00
S	20.67	1.53	R	8.67	0.58	R	11.67
S	20.67	1.15	R	9.00	1.00	R	10.67
S	21.67	0.58	R	7.67	1.15	R	12.67
S	21.00	1.73	R	7.67	0.58	R	12.00
S	20.67	1.53	R	9.33	1.15	R	11.67
S	21.00	1.00	R	8.00	1.00	R	11.33
S	20.00	1.73	R	9.00	0.00	R	12.00
S	21.00	1.00	R	8.67	1.53	R	12.33
S	19.67	1.15	I	14.00	1.00	R	11.00
S	20.33	0.58	I	15.00	1.00	R	12.00
S	20.33	1.53	I	15.33	1.15	R	11.67
S	19.33	0.58	I	14.67	1.53	R	11.00
S	20.67	1.53	I	15.33	0.58	R	12.00
S	21.00	1.00	I	14.67	1.53	R	12.33
S	22.00	0.00	I	14.00	1.00	R	12.00
S	20.67	0.58	S	19.00	1.73	R	11.00
S	21.33	0.58	S	18.33	1.53	R	12.33
S	21.33	1.15	S	18.33	0.58	R	11.00
S	21.00	1.00	S	18.67	1.53	R	11.00
S	20.00	1.00	S	18.33	1.15	R	10.00
S	21.00	1.73	S	17.33	0.58	R	12.33
S	19.33	0.58	S	18.33	0.58	R	11.67
S	20.00	0.00	S	18.67	1.53	R	11.33
S	19.67	0.58	S	19.67	0.58	R	11.67
S	21.00	1.73	S	17.67	1.15	I	18.33
S	21.33	1.15	S	19.00	1.00	I	17.00
S	21.67	0.58	S	18.67	1.53	I	18.67
S	21.00	1.00	S	19.33	0.58	I	16.67
S	22.00	0.00	S	19.00	0.00	I	19.33
S	19.33	0.58	S	17.67	1.15	I	18.67
S	20.67	0.58	S	18.67	1.15	I	17.67
S	20.00	0.00	S	18.33	1.15	I	16.00
S	20.33	1.53	S	18.67	1.53	I	17.67

S	20.00	1.00	S	17.67	0.58	I	17.33
S	19.33	0.58	S	18.67	1.53	I	19.00
S	20.67	1.15	S	18.00	1.73	I	18.67
S	20.67	0.58	S	18.00	1.00	I	19.33
S	20.00	1.73	S	17.67	0.58	I	17.33
S	19.67	1.15	S	18.00	0.00	I	20.33
S	21.33	0.58	S	17.67	1.15	I	18.33
S	19.67	1.15	S	19.00	1.73	S	24.67
S	20.00	1.73	S	20.00	0.00	S	25.33
S	20.33	1.15	S	19.67	0.58	S	22.67
S	21.00	1.00	S	17.33	0.58	S	22.67
S	19.33	0.58	S	18.33	1.15	S	22.67
S	19.67	1.15	S	17.67	1.15	S	24.33
S	19.67	0.58	S	19.00	1.00	S	23.00
S	20.67	1.53	S	18.33	1.53	S	24.67
S	21.00	1.00	S	17.67	1.15	S	25.00
S	21.33	1.15	S	19.33	1.15	S	26.00
S	20.33	1.53	S	19.33	0.58	S	22.67
S	21.00	0.00	S	17.67	0.58	S	23.00
S	19.33	0.58	S	18.67	1.53	S	23.33
S	20.33	1.15	S	18.67	0.58	S	23.33
S	20.33	1.15	S	18.33	1.53	S	24.33
S	20.67	1.53	S	19.33	0.58	S	24.33
S	19.33	0.58	S	18.33	1.15	S	24.33
S	20.33	1.53	S	18.33	1.53	S	23.67
S	20.67	1.15	S	18.33	1.53	S	23.67
S	21.00	1.73	S	18.00	0.00	S	25.00
S	21.00	1.00	S	17.67	0.58	S	23.67
S	20.33	1.15	S	18.33	1.15	S	24.67
S	20.67	0.58	S	19.33	1.15	S	25.67
S	21.33	1.15	S	18.00	1.73	S	24.67
S	21.00	1.00	S	18.00	1.00	S	25.00
S	20.67	1.15	S	18.33	1.53	S	24.00
S	21.00	1.73	S	19.00	1.73	S	23.33
S	21.33	1.15	S	19.33	1.15	S	24.67
S	21.33	0.58	S	19.00	1.00	S	22.67
S	20.67	0.58	S	18.67	0.58	S	24.00
S	20.00	1.00	S	18.67	1.53	S	24.00
S	20.00	1.00	S	19.67	0.58	S	25.00
S	19.67	0.58	S	18.67	1.53	S	22.67

	Ciprofloxacin			kanamycin			Ge
SD	Interpretation	Mean	SD	Interpretation	Mean	SD	Interpretation
0.58	R	13.67	1.53	R	12.33	0.58	R
1.53	R	15.00	0.00	R	12.00	1.00	R
0.00	R	12.67	0.58	R	11.33	0.58	R
0.58	R	13.33	1.15	R	12.33	0.58	R
1.15	R	13.67	0.58	R	12.33	0.58	R
0.58	R	14.00	1.00	R	12.00	1.73	R
1.00	R	13.00	1.73	R	11.00	1.00	R
1.15	R	13.67	0.58	R	11.33	1.15	R
0.58	R	13.67	1.53	R	12.00	1.00	R
1.53	R	14.33	1.15	R	11.33	0.58	R
0.00	R	14.33	1.15	R	12.00	1.00	R
0.58	R	13.33	0.58	R	11.33	1.15	R
1.73	R	13.67	1.53	R	11.00	1.00	R
1.15	R	14.00	1.73	R	12.33	0.58	R
1.15	R	14.33	0.58	R	11.67	1.53	R
0.58	R	13.67	1.53	R	10.67	1.15	R
1.53	R	13.67	1.53	R	11.67	0.58	R
0.58	R	13.67	0.58	R	12.33	1.15	R
1.00	R	13.33	0.58	R	11.33	0.58	R
0.58	R	12.33	0.58	R	12.33	1.15	R
0.58	R	12.67	0.58	R	11.67	1.53	R
1.15	R	14.00	1.73	R	12.33	1.15	R
1.73	R	13.67	1.53	R	11.00	1.73	R
0.58	R	13.00	0.00	R	11.67	0.58	R
1.00	R	12.33	0.58	R	11.67	0.58	R
1.00	R	14.33	0.58	R	10.67	1.15	R
1.53	R	14.00	1.00	R	11.33	1.53	R
1.53	R	14.00	1.00	R	11.67	1.15	I
1.73	R	12.33	0.58	I	16.00	1.00	I
1.00	R	12.33	0.58	I	15.67	0.58	I
1.15	R	15.00	0.00	I	15.67	1.15	I
1.00	R	12.67	1.15	I	16.33	0.58	I
1.53	R	15.00	0.00	I	16.67	0.58	I
1.15	R	13.33	0.58	I	16.00	1.00	I
1.15	R	13.67	0.58	I	16.67	0.58	I
0.00	R	13.33	1.15	I	15.67	1.15	I
0.58	R	13.67	1.15	I	16.00	0.00	I
0.58	R	13.33	1.53	I	15.33	0.58	I
0.58	R	13.00	1.00	I	15.67	0.58	I
1.00	R	13.67	1.53	I	15.33	0.58	I
1.00	R	14.00	1.73	I	15.33	0.58	S
1.53	R	13.33	1.53	I	16.67	0.58	S
0.58	R	13.67	1.53	I	15.33	0.58	S
0.58	R	14.00	1.00	I	16.67	0.58	S
1.00	R	14.00	1.00	I	16.00	1.00	S

0.58	R	13.33	0.58	I	16.33	1.15	S
0.58	R	13.67	1.53	I	16.33	1.15	S
1.00	R	13.33	1.53	I	16.00	1.00	S
1.00	R	13.00	1.00	I	15.00	0.00	S
1.53	R	13.67	1.53	I	15.67	1.15	S
1.00	R	12.67	0.58	I	16.00	1.00	S
0.58	R	13.00	1.73	I	15.67	0.58	S
1.15	R	13.00	1.00	I	15.67	0.58	S
0.58	R	12.67	0.58	I	15.33	0.58	S
1.53	R	14.00	1.00	I	15.67	0.58	S
1.73	R	13.67	1.53	I	17.00	0.00	S
1.15	R	14.67	0.58	I	15.33	0.58	S
0.58	R	13.33	1.15	I	16.67	0.58	S
1.00	R	13.00	1.00	I	16.00	1.00	S
1.15	I	17.00	1.73	I	16.33	1.15	S
0.58	I	17.67	0.58	I	15.33	0.58	S
0.58	I	18.67	0.58	I	16.00	1.00	S
1.73	I	17.00	1.00	I	16.00	1.00	S
1.15	I	16.67	1.15	I	16.33	1.15	S
1.53	I	18.00	1.73	I	16.00	1.00	S
1.00	I	18.00	1.73	I	16.33	0.58	S
1.15	I	17.67	1.53	I	15.33	0.58	S
1.00	I	18.00	1.00	I	15.67	1.15	S
1.73	I	18.00	1.00	I	16.00	0.00	S
1.53	I	17.67	1.53	I	16.00	1.00	S
0.00	I	16.33	0.58	I	15.00	0.00	S
1.00	I	17.67	1.53	I	16.67	0.58	S
1.15	I	18.00	1.00	I	15.67	0.58	S
1.00	I	18.00	1.00	S	21.67	1.15	S
1.00	I	18.00	1.00	S	21.67	0.58	S
0.58	I	17.00	1.00	S	20.33	1.53	S
1.73	I	17.33	1.53	S	22.67	0.58	S
1.00	I	17.33	1.53	S	21.67	0.58	S
0.00	I	16.67	1.15	S	20.33	2.31	S
1.15	I	16.67	0.58	S	20.33	2.31	S
1.53	I	17.67	1.53	S	21.00	1.00	S
0.58	I	17.00	1.00	S	20.67	2.08	S
1.53	I	17.33	0.58	S	21.67	1.53	S
0.58	I	18.33	0.58	S	21.67	1.53	S
1.00	I	17.33	0.58	S	22.67	0.58	S
3.21	I	18.00	1.00	S	21.00	1.00	S
0.58	I	18.00	1.00	S	21.33	1.53	S
1.53	I	17.33	1.53	S	21.67	1.15	S
2.52	I	18.67	0.58	S	22.00	1.73	S
1.15	I	17.00	1.00	S	20.33	2.31	S
1.73	I	17.00	1.00	S	21.00	1.00	S
3.06	I	17.67	1.15	S	21.33	1.15	S

3.21	I	17.67	0.58	S	21.33	2.08	S
1.00	I	18.33	0.58	S	22.67	0.58	S
2.52	I	19.00	0.00	S	20.00	1.00	S
1.53	I	16.67	0.58	S	22.33	0.58	S
2.31	I	17.33	1.53	S	20.33	1.53	S
1.15	I	18.33	0.58	S	20.00	1.73	S
1.53	I	17.00	1.73	S	21.33	1.53	S
1.15	S	23.67	1.53	S	20.67	2.08	S
0.58	S	21.00	1.73	S	21.67	1.53	S
0.58	S	23.00	2.65	S	19.67	1.15	S
1.15	S	23.33	2.08	S	22.00	1.00	S
1.15	S	23.67	1.15	S	19.67	1.15	S
2.08	S	21.67	0.58	S	21.00	1.73	S
1.73	S	22.67	2.08	S	21.67	1.15	S
1.53	S	22.33	2.31	S	19.00	0.00	S
0.00	S	22.00	2.65	S	20.00	1.73	S
0.00	S	22.00	2.00	S	21.33	1.53	S
0.58	S	23.67	1.53	S	20.33	0.58	S
1.73	S	24.00	1.00	S	20.00	1.00	S
2.31	S	23.67	1.15	S	21.33	1.53	S
1.15	S	22.67	2.31	S	20.67	1.15	S
1.53	S	24.33	1.15	S	20.00	1.00	S
1.15	S	21.67	1.53	S	19.67	0.58	S
1.53	S	24.33	0.58	S	21.00	1.73	S
2.08	S	21.33	1.15	S	21.33	1.15	S
1.53	S	22.67	1.15	S	20.67	1.53	S
1.00	S	23.33	2.89	S	21.00	1.73	S
2.08	S	21.33	1.15	S	21.00	1.73	S
1.15	S	23.33	2.08	S	21.00	1.00	S
0.58	S	23.00	2.00	S	23.00	0.00	S
1.53	S	21.33	2.31	S	20.33	2.31	S
1.00	S	21.67	1.53	S	20.67	2.08	S
1.00	S	22.00	2.00	S	20.67	2.08	S
1.53	S	24.33	1.15	S	21.00	1.00	S
2.31	S	24.33	1.15	S	20.67	1.53	S
1.15	S	21.67	1.53	S	21.33	1.53	S
1.73	S	23.33	2.08	S	20.33	0.58	S
2.00	S	21.00	1.00	S	20.00	1.00	S
1.00	S	21.33	0.58	S	20.67	2.08	S
0.58	S	22.33	2.08	S	20.33	0.58	S

ntamycin		Cefepime			Cefoxitin		
Mean	SD	Interpretation	Mean	SD	Interpretation	Mean	SD
9.67	0.58	R	17.00	1.00	R	12.33	1.15
9.67	0.58	R	17.00	1.00	R	10.33	0.58
9.67	1.15	R	16.67	1.15	R	11.67	1.53
10.67	1.15	R	16.33	0.58	R	12.00	1.73
11.00	1.00	R	17.33	1.15	R	13.00	1.73
10.00	0.00	R	16.00	1.73	R	12.33	2.08
10.33	1.53	R	16.00	1.00	R	11.67	2.08
9.33	0.58	R	16.33	1.15	R	11.67	2.08
10.00	1.00	R	16.67	1.53	R	11.33	0.58
11.00	1.00	R	15.00	0.00	R	12.33	1.53
10.00	1.00	R	16.33	1.15	R	13.00	1.00
9.33	0.58	R	17.67	0.58	R	12.00	1.73
11.00	1.00	R	16.67	1.15	R	12.33	2.08
10.67	1.53	R	16.00	1.73	R	10.67	1.15
10.33	1.15	R	16.33	1.15	R	11.67	1.53
10.00	1.73	R	16.00	1.00	R	10.67	0.58
11.00	1.73	R	16.00	1.73	R	11.67	0.58
11.67	0.58	R	16.00	1.00	R	12.00	1.00
11.67	0.58	R	17.67	0.58	R	11.33	1.53
10.33	1.53	R	17.67	0.58	R	11.00	1.00
10.67	1.53	R	15.67	1.15	R	12.00	1.00
10.67	1.15	R	17.00	1.00	I	16.00	0.00
10.33	1.53	R	16.33	1.15	I	17.33	1.15
10.67	1.53	R	16.67	0.58	I	15.67	0.58
11.00	1.00	R	16.67	1.53	I	16.00	1.00
10.33	1.53	R	15.33	0.58	I	16.00	1.00
10.33	1.53	R	16.67	1.15	I	16.00	1.73
14.67	0.58	R	16.67	0.58	I	16.00	1.00
13.33	0.58	R	17.67	0.58	I	16.00	1.00
13.67	1.15	R	16.33	1.53	I	17.00	1.00
14.67	0.58	R	17.67	0.58	I	16.33	1.53
14.00	1.00	R	16.00	1.73	I	17.33	0.58
14.00	1.00	R	17.00	1.00	I	16.00	1.00
13.67	1.15	R	16.67	1.53	I	16.33	0.58
14.00	1.00	R	16.67	1.53	I	17.33	1.15
13.33	0.58	R	16.33	1.53	S	22.33	0.58
13.67	0.58	R	16.00	1.00	S	19.67	1.15
14.33	0.58	R	17.00	1.00	S	21.67	2.08
13.33	0.58	R	15.33	0.58	S	21.00	2.00
14.33	0.58	R	16.33	1.15	S	21.33	2.08
18.33	1.53	R	16.33	1.53	S	21.33	1.53
17.67	1.53	R	17.00	1.00	S	22.00	2.00
19.67	0.58	R	17.00	1.73	S	22.33	2.89
18.00	1.00	R	16.00	1.00	S	21.33	2.52
17.00	1.00	R	16.33	1.53	S	22.00	0.00

16.67	0.58	R	16.33	0.58	S	22.67	1.53
17.67	1.53	R	17.00	1.73	S	21.33	2.08
17.33	0.58	R	17.33	0.58	S	22.33	1.15
18.67	1.53	R	16.00	1.00	S	20.67	0.58
19.00	1.73	R	15.00	0.00	S	20.67	0.58
17.67	0.58	R	16.67	0.58	S	23.00	1.00
18.67	1.53	R	16.67	1.15	S	22.00	2.00
16.67	0.58	R	17.33	1.15	S	21.33	2.08
18.00	1.73	R	15.33	0.58	S	22.33	2.89
18.00	1.73	R	16.33	0.58	S	23.33	0.58
17.00	1.73	R	17.00	1.00	S	22.67	0.58
19.00	0.00	R	17.00	1.00	S	20.33	2.31
17.33	0.58	R	15.67	1.15	S	22.33	2.08
17.33	1.53	R	16.67	1.15	S	22.33	0.58
17.67	1.53	R	16.33	1.15	S	22.67	0.58
18.00	1.00	R	17.00	1.73	S	20.67	2.08
17.67	0.58	R	16.33	1.15	S	23.33	1.15
18.67	1.53	R	18.00	0.00	S	20.67	2.08
17.67	2.08	R	16.00	1.00	S	20.00	1.00
17.67	1.53	R	17.67	0.58	S	21.33	2.08
18.33	1.15	R	15.67	1.15	S	20.33	1.53
17.33	1.53	R	17.00	1.00	S	21.33	2.08
17.00	1.73	R	16.67	0.58	S	20.67	0.58
16.67	0.58	R	17.00	1.00	S	21.33	2.52
17.33	2.31	R	16.00	1.73	S	22.33	2.08
17.00	1.00	R	16.33	1.53	S	22.67	0.58
17.67	1.15	I	23.67	0.58	S	22.33	0.58
19.00	1.73	I	22.00	2.00	S	21.00	2.00
18.67	1.15	I	22.33	2.08	S	21.33	1.53
17.00	1.73	I	21.67	0.58	S	21.33	0.58
18.33	1.15	I	22.33	1.15	S	22.00	1.00
17.00	1.73	I	21.00	1.73	S	21.67	2.08
17.33	1.15	I	22.00	2.65	S	20.00	1.00
18.33	1.53	I	20.67	2.08	S	22.00	0.00
17.33	2.31	I	21.67	1.53	S	21.67	2.08
18.67	0.58	I	21.00	2.00	S	19.67	0.58
19.67	0.58	I	21.67	2.08	S	20.67	0.58
18.00	1.73	I	21.00	2.65	S	21.00	2.65
18.00	2.00	I	22.00	1.00	S	22.33	1.53
16.67	1.15	I	20.67	1.53	S	20.67	1.53
17.33	1.53	I	21.67	2.31	S	22.33	2.89
18.33	1.15	S	27.00	1.00	S	20.67	0.58
18.33	1.53	S	27.67	2.08	S	22.00	0.00
18.00	2.00	S	26.67	0.58	S	22.00	1.73
19.00	1.00	S	27.33	1.53	S	22.00	2.00
18.33	1.53	S	28.33	2.08	S	21.67	1.53
17.33	0.58	S	29.00	1.73	S	22.67	0.58

18.00	1.00	S	27.67	0.58	S	22.67	2.31
19.00	1.00	S	28.67	0.58	S	19.33	0.58
18.33	0.58	S	28.00	1.00	S	19.67	1.15
17.33	2.31	S	27.33	1.53	S	21.33	1.15
18.67	1.15	S	27.67	1.15	S	20.33	1.53
17.00	1.00	S	27.33	1.53	S	21.33	2.08
19.00	1.73	S	30.00	0.00	S	20.67	1.15
18.67	2.31	S	29.00	1.73	S	22.00	2.65
18.00	2.00	S	29.33	0.58	S	20.67	1.53
17.67	1.15	S	27.67	1.15	S	21.00	2.65
17.00	1.73	S	26.67	1.15	S	20.00	1.73
17.33	1.53	S	28.67	2.31	S	20.67	1.53
17.67	2.08	S	27.33	1.53	S	20.00	1.00
17.67	1.15	S	30.00	0.00	S	21.67	1.53
17.67	1.53	S	29.33	0.58	S	21.00	1.00
17.67	0.58	S	28.67	1.53	S	21.67	2.52
19.67	0.58	S	27.67	2.08	S	21.00	2.00
18.67	1.15	S	27.33	1.53	S	22.33	1.53
18.67	1.15	S	29.00	1.00	S	21.67	2.08
17.67	0.58	S	29.00	1.73	S	20.00	1.00
17.33	1.53	S	28.00	1.73	S	21.00	2.65
19.33	1.15	S	27.67	0.58	S	21.00	1.73
18.00	2.00	S	27.67	1.53	S	23.00	1.00
17.00	1.73	S	28.33	1.15	S	19.67	0.58
17.33	1.53	S	27.67	2.08	S	22.33	1.53
19.00	1.00	S	29.00	1.73	S	22.33	1.53
19.00	1.00	S	28.00	1.00	S	21.00	1.00
17.67	2.08	S	28.33	2.08	S	22.00	1.00
18.33	0.58	S	27.33	0.58	S	23.33	1.15
18.67	1.15	S	28.00	1.73	S	21.00	2.65
19.00	1.00	S	27.67	1.53	S	21.00	2.65
18.00	1.00	S	28.00	1.00	S	23.33	0.58
18.67	1.53	S	27.33	0.58	S	23.33	0.58
17.33	1.53	S	28.00	1.00	S	21.00	1.73
18.33	2.08	S	26.67	1.15	S	20.33	1.53
17.67	1.53	S	26.33	0.58	S	20.33	1.15
18.00	2.00	S	27.00	1.73	S	20.33	1.15
19.33	0.58	S	26.67	0.58	S	21.33	2.08
17.33	1.15	S	28.67	1.53	S	22.67	1.15
18.33	1.53	S	26.33	0.58	S	23.33	1.15

Cephalexin			Fosfomycin			Chloramphenico	
Interpretation	Mean	SD	Interpretation	Mean	SD	Interpretation	Mean
R	10.67	0.58	R	10.00	1.73	R	12.00
R	10.33	0.58	R	10.33	1.53	R	10.00
R	11.00	1.73	R	10.33	1.53	R	10.67
R	10.33	1.53	R	11.00	1.00	R	11.33
R	9.67	1.15	R	11.33	0.58	R	10.33
R	11.33	0.58	R	10.00	1.73	R	10.33
R	9.67	0.58	R	10.67	1.53	R	10.67
R	10.67	1.15	R	10.67	1.53	R	10.00
R	10.33	1.15	I	15.33	0.58	R	10.67
R	10.00	1.00	I	15.33	0.58	R	10.33
R	11.00	1.00	I	13.33	0.58	R	11.33
R	10.00	1.00	S	30.33	0.58	R	10.33
R	10.67	0.58	S	28.67	1.53	R	10.00
R	9.67	0.58	S	29.67	1.53	R	10.33
R	9.67	0.58	S	28.33	0.58	R	11.33
R	9.67	0.58	S	29.00	2.00	R	11.00
R	9.67	0.58	S	29.33	0.58	R	9.67
R	11.67	0.58	S	27.67	0.58	R	10.00
R	10.67	1.53	S	27.33	0.58	R	10.00
R	10.00	1.00	S	28.00	1.73	R	11.33
R	10.67	1.53	S	28.67	1.53	R	11.33
R	9.67	0.58	S	28.67	2.08	I	15.67
R	11.00	1.73	S	27.33	0.58	I	14.33
R	11.00	1.00	S	28.33	1.53	I	14.67
R	9.67	1.15	S	30.00	1.00	I	14.00
R	9.33	0.58	S	29.00	1.00	I	14.67
R	10.33	1.53	S	30.67	0.58	I	14.33
R	11.00	1.73	S	29.33	1.53	I	15.00
R	11.33	0.58	S	27.67	1.15	I	14.00
R	9.67	1.15	S	29.00	2.00	I	15.00
R	10.67	0.58	S	29.00	1.00	S	29.33
R	10.33	1.15	S	28.00	1.00	S	28.67
R	10.67	0.58	S	29.00	1.00	S	27.67
R	11.67	0.58	S	28.67	1.15	S	29.00
R	10.33	1.53	S	28.67	1.15	S	30.33
R	11.00	1.00	S	29.00	1.00	S	30.67
R	10.33	1.15	S	30.33	1.15	S	28.67
R	9.67	0.58	S	29.67	1.53	S	28.33
R	11.00	1.00	S	28.67	1.15	S	30.33
R	10.00	1.00	S	29.33	2.08	S	29.33
R	11.33	0.58	S	30.00	1.00	S	29.67
R	10.33	1.15	S	28.67	2.08	S	27.33
R	9.67	1.15	S	29.67	0.58	S	29.00
R	11.33	0.58	S	29.33	2.08	S	29.00
R	11.67	0.58	S	30.33	0.58	S	29.67

R	10.00	1.73	S	29.67	1.53	S	29.67
R	11.00	1.00	S	29.00	2.00	S	29.00
R	10.00	1.00	S	29.00	1.00	S	28.33
R	10.00	1.00	S	28.67	2.08	S	28.67
R	11.00	1.00	S	28.67	2.08	S	28.67
R	11.00	1.73	S	28.33	0.58	S	29.67
R	9.67	0.58	S	29.00	1.73	S	29.00
R	10.00	1.00	S	30.33	0.58	S	29.00
R	10.67	1.53	S	27.67	1.15	S	30.67
R	10.00	1.00	S	27.00	0.00	S	29.00
R	11.00	1.73	S	29.33	2.08	S	27.67
R	11.00	1.00	S	29.00	1.73	S	28.33
R	11.00	1.00	S	28.33	1.53	S	29.00
R	10.00	1.73	S	30.00	1.73	S	28.00
R	10.33	0.58	S	28.00	0.00	S	28.00
R	10.67	1.15	S	29.67	0.58	S	29.00
R	11.00	1.00	S	28.00	1.00	S	28.67
R	10.67	0.58	S	28.33	0.58	S	29.33
R	10.67	1.53	S	29.00	1.73	S	28.33
R	11.00	1.73	S	29.00	1.00	S	30.33
R	10.33	0.58	S	29.33	1.53	S	28.67
R	10.33	1.53	S	28.33	0.58	S	30.00
R	10.33	1.53	S	29.00	1.00	S	28.00
R	10.00	1.00	S	27.67	1.15	S	28.00
R	9.33	0.58	S	29.00	1.73	S	28.67
R	10.00	1.00	S	30.00	1.00	S	29.67
R	10.33	1.53	S	29.00	2.00	S	28.67
R	9.67	0.58	S	28.00	1.73	S	29.33
R	10.67	1.15	S	30.00	1.00	S	29.00
R	10.67	1.53	S	29.00	1.00	S	30.33
R	10.33	1.53	S	30.00	1.73	S	29.67
R	10.00	1.00	S	30.33	0.58	S	29.67
R	11.33	0.58	S	30.33	0.58	S	29.33
R	9.67	1.15	S	30.00	1.00	S	28.33
R	11.00	1.00	S	29.00	1.00	S	29.00
R	9.67	0.58	S	27.00	0.00	S	29.67
R	10.67	0.58	S	28.00	1.00	S	28.33
R	11.00	1.73	S	29.33	2.08	S	28.67
R	9.67	1.15	S	29.67	0.58	S	29.33
R	10.00	1.73	S	30.67	0.58	S	29.00
R	10.67	1.53	S	29.33	1.53	S	27.67
R	11.00	1.00	S	29.00	1.73	S	29.00
R	10.00	1.73	S	29.67	1.53	S	27.67
R	10.67	1.53	S	30.00	0.00	S	29.33
R	10.00	1.00	S	28.67	0.58	S	28.67
R	11.00	1.00	S	28.33	0.58	S	29.67
R	11.67	0.58	S	29.00	1.00	S	29.33

R	11.33	1.15	S	30.00	1.00	S	28.67
R	10.33	1.53	S	27.33	0.58	S	28.67
R	10.33	1.15	S	28.67	2.08	S	29.67
R	10.33	1.53	S	30.00	1.73	S	28.33
R	10.33	1.53	S	30.33	1.15	S	28.33
R	9.00	0.00	S	29.00	1.00	S	29.67
R	11.67	0.58	S	28.67	0.58	S	27.33
R	10.33	1.53	S	28.67	1.53	S	30.00
S	30.00	1.00	S	28.67	0.58	S	28.67
S	29.67	1.53	S	28.00	1.73	S	28.33
S	29.67	2.31	S	30.67	0.58	S	29.67
S	30.33	0.58	S	28.33	0.58	S	29.33
S	29.67	0.58	S	29.67	2.31	S	29.67
S	29.33	2.08	S	28.33	1.15	S	30.67
S	28.33	0.58	S	29.33	1.15	S	28.67
S	30.67	0.58	S	30.00	1.00	S	28.67
S	28.33	0.58	S	29.00	2.00	S	28.00
S	28.33	0.58	S	29.00	1.00	S	28.33
S	29.33	1.15	S	30.33	0.58	S	29.33
S	28.67	1.53	S	30.00	1.73	S	27.67
S	30.33	0.58	S	29.33	2.08	S	27.67
S	28.00	1.00	S	29.00	1.00	S	28.67
S	27.67	1.15	S	28.33	1.53	S	28.67
S	29.33	2.08	S	28.67	1.15	S	29.00
S	27.67	0.58	S	28.67	1.53	S	28.67
S	28.00	1.73	S	29.00	2.00	S	29.00
S	28.33	1.15	S	29.00	1.73	S	29.00
S	29.00	2.00	S	29.00	1.00	S	29.00
S	29.00	0.00	S	29.67	2.31	S	28.00
S	29.67	1.15	S	29.67	2.31	S	29.33
S	28.67	2.08	S	28.33	1.53	S	28.67
S	30.33	1.15	S	30.00	1.73	S	28.67
S	30.00	1.00	S	29.33	2.08	S	29.33
S	28.00	1.73	S	28.67	1.53	S	29.67
S	28.67	0.58	S	30.00	1.00	S	29.00
S	30.33	1.15	S	29.67	2.31	S	29.33
S	28.33	1.15	S	27.67	1.15	S	29.67
S	28.67	1.53	S	29.00	2.00	S	29.00
S	29.33	1.53	S	27.67	1.15	S	28.00
S	30.00	1.00	S	29.67	1.53	S	29.67

l	Florfenicol		
SD	Interpretation	Mean	SD
0.00	R	8.00	0.00
1.00	R	8.67	0.58
1.53	R	9.00	0.00
1.15	R	9.67	0.58
0.58	R	9.67	0.58
1.53	R	9.00	1.00
1.53	R	9.00	1.00
1.73	R	9.00	1.00
0.58	R	9.33	0.58
1.53	R	9.33	0.58
1.15	R	9.00	1.00
1.53	R	10.00	0.00
1.00	R	8.67	0.58
1.15	R	9.00	1.00
0.58	I	15.00	1.00
1.00	S	20.67	3.79
0.58	S	23.67	5.51
1.00	S	21.00	2.65
1.73	S	24.00	3.61
1.15	S	29.00	0.00
1.15	S	26.00	0.00
0.58	S	21.00	3.61
1.15	S	21.33	3.51
1.53	S	24.00	5.57
1.00	S	25.67	4.04
1.15	S	25.00	7.00
1.15	S	24.33	5.03
1.73	S	28.00	3.46
1.73	S	26.00	4.36
1.00	S	23.33	2.08
1.53	S	21.67	3.06
2.08	S	21.00	3.46
1.15	S	22.00	3.00
2.00	S	24.00	1.00
0.58	S	22.00	3.61
0.58	S	24.33	6.35
2.08	S	25.33	3.06
0.58	S	21.67	2.31
0.58	S	24.33	4.04
2.08	S	20.67	3.21
2.31	S	18.00	0.00
0.58	S	25.33	3.51
2.00	S	21.00	4.00
1.00	S	21.00	3.61
2.31	S	25.67	5.86

1.15	S	24.33	5.51
1.00	S	20.33	2.31
1.53	S	25.00	3.46
2.08	S	24.67	6.11
2.08	S	23.67	5.51
2.31	S	26.33	2.08
1.00	S	23.00	0.00
1.73	S	21.33	4.51
0.58	S	23.33	2.31
2.00	S	25.00	6.08
1.15	S	25.00	2.65
1.15	S	24.33	5.69
2.00	S	22.67	4.51
1.00	S	21.33	5.86
1.73	S	21.33	1.53
1.00	S	24.00	1.00
2.08	S	22.67	5.13
1.53	S	26.67	4.93
1.53	S	25.00	3.61
0.58	S	22.67	5.51
1.15	S	23.00	1.73
1.00	S	21.67	7.23
1.00	S	20.33	4.16
1.73	S	28.00	2.00
1.53	S	27.67	2.31
1.53	S	22.00	3.46
0.58	S	22.33	4.73
1.53	S	26.33	3.51
1.73	S	23.67	4.51
0.58	S	22.33	0.58
0.58	S	20.67	2.89
0.58	S	23.67	2.52
2.08	S	23.00	5.57
2.31	S	22.00	2.65
1.00	S	19.00	2.65
0.58	S	20.00	2.65
1.53	S	20.67	2.08
1.53	S	24.67	2.08
2.08	S	23.67	3.79
1.00	S	19.33	2.08
1.15	S	22.67	2.08
1.00	S	25.00	6.24
0.58	S	23.67	3.79
1.53	S	23.00	4.36
2.08	S	26.00	3.00
1.53	S	25.00	5.20
1.53	S	28.67	2.31

1.53	S	23.67	5.13
1.15	S	18.33	2.31
0.58	S	22.33	3.51
1.53	S	24.00	3.61
0.58	S	21.33	5.13
2.31	S	24.67	5.77
0.58	S	21.33	2.08
1.73	S	25.33	3.21
1.53	S	25.00	4.58
1.15	S	26.00	4.36
1.53	S	21.00	6.08
1.53	S	21.67	5.03
1.53	S	26.67	1.53
0.58	S	25.67	5.77
2.08	S	23.00	2.65
2.08	S	27.00	2.65
1.00	S	23.67	6.03
1.53	S	24.67	6.11
1.53	S	20.67	3.79
0.58	S	25.67	2.52
0.58	S	24.00	5.00
1.53	S	26.00	5.29
2.08	S	26.33	6.35
2.00	S	23.67	5.51
1.53	S	25.67	2.31
1.73	S	24.00	1.00
2.00	S	25.00	3.61
1.73	S	24.33	1.15
0.00	S	23.67	5.13
2.08	S	26.00	6.08
1.53	S	22.67	5.51
1.53	S	25.00	4.36
1.15	S	26.67	2.08
2.31	S	24.00	5.29
2.00	S	25.67	4.04
1.53	S	24.67	5.51
1.53	S	26.33	2.08
2.00	S	19.67	2.52
1.73	S	23.00	4.36
1.53	S	20.67	3.79
