# Supplementary material for: Colistin-resistance genes in Escherichia coli isolated from patients with urinary tract infections
Source: PLoS One. 2024 Jun 12;19(6):e0305431. doi: 10.1371/journal.pone.0305431 (PMC11168671; doi:10.1371/journal.pone.0305431)
Supplement: S1 Raw images — (PDF) [file pone.0305431.s002.pdf]

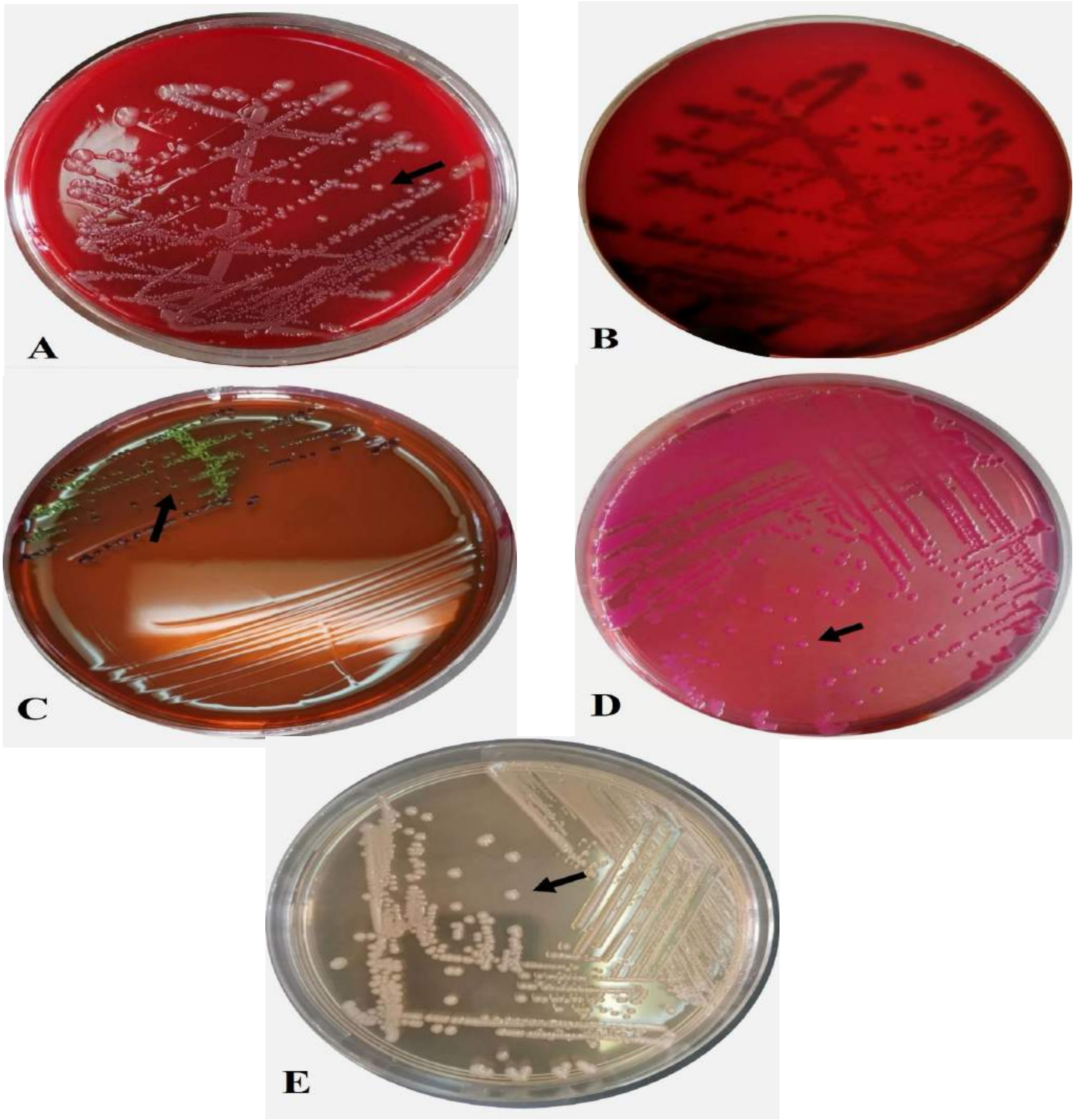

Figure 1 | *E. coli* colony morphology on different types of agar media. (A, B) *E. coli* on blood agar. (C) *E. coli* on EMB agar. (D) *E. coli* on MacConkey agar. (E) *E. coli* on Nutrient Agar.

Figure 2 | Electrophoresis for *E. coli uspA* gene. Lane M: 100bp ladder; lane 1 to 10: samples; lane P: positive, and N: negative controls**.**

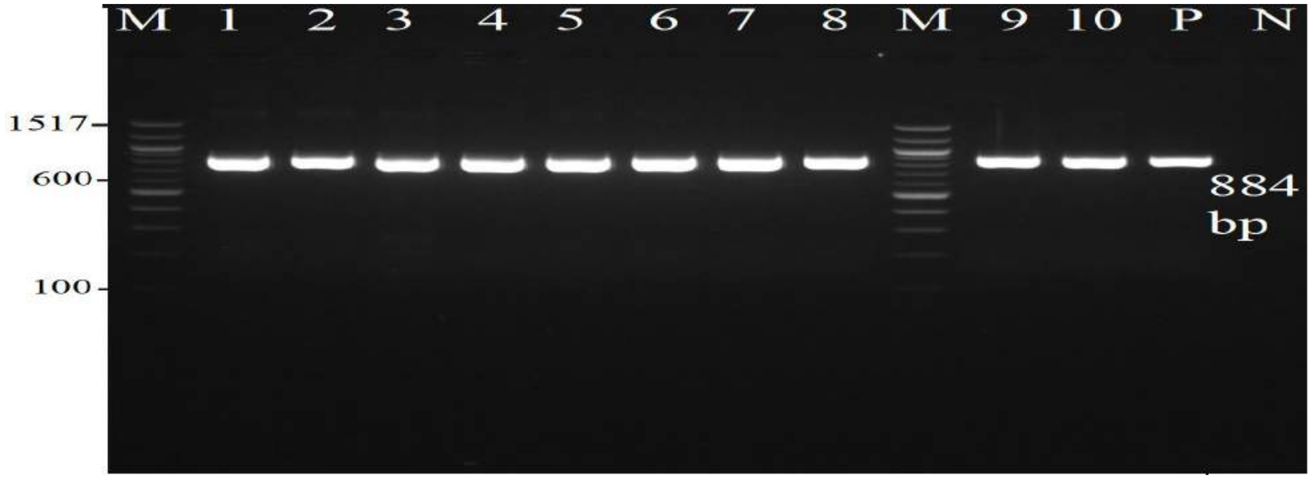

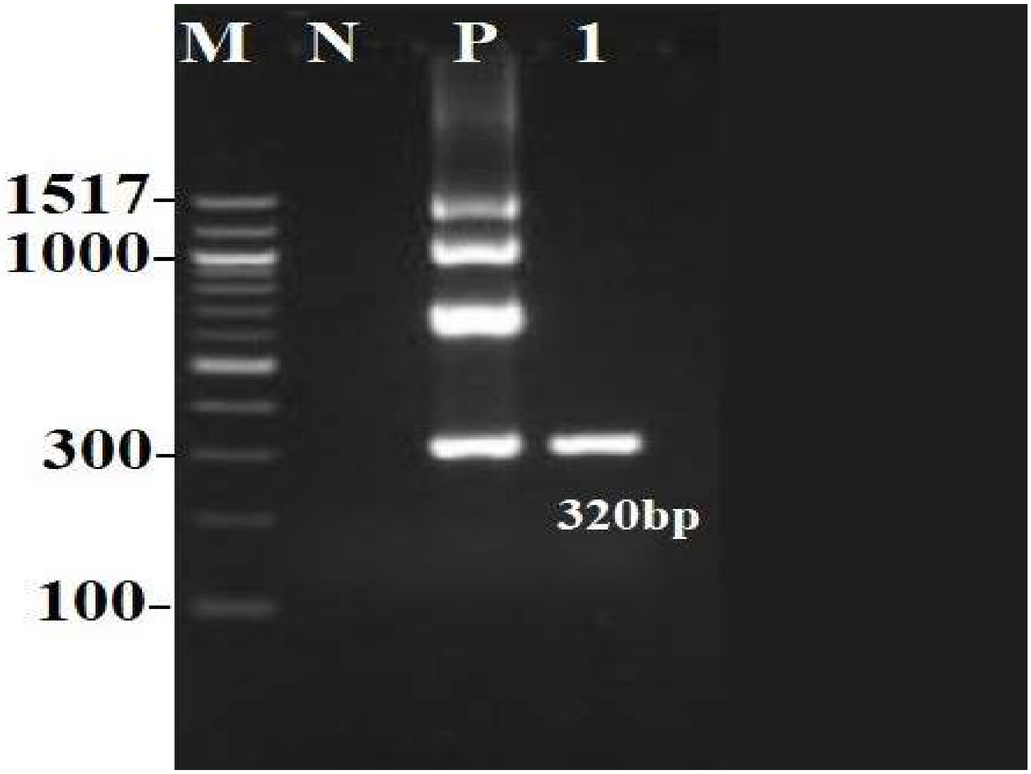

Figure 3 | Electrophoresis for a single *E. coli* isolate carries *MCR*-1gene.

Lane M: 100bp ladder; lane 1: A sample *E. coli* isolate carries *MCR*-1gene; lane P: positive for (*MCR* 1-5) except *MCR* -3, and N: negative controls**.**

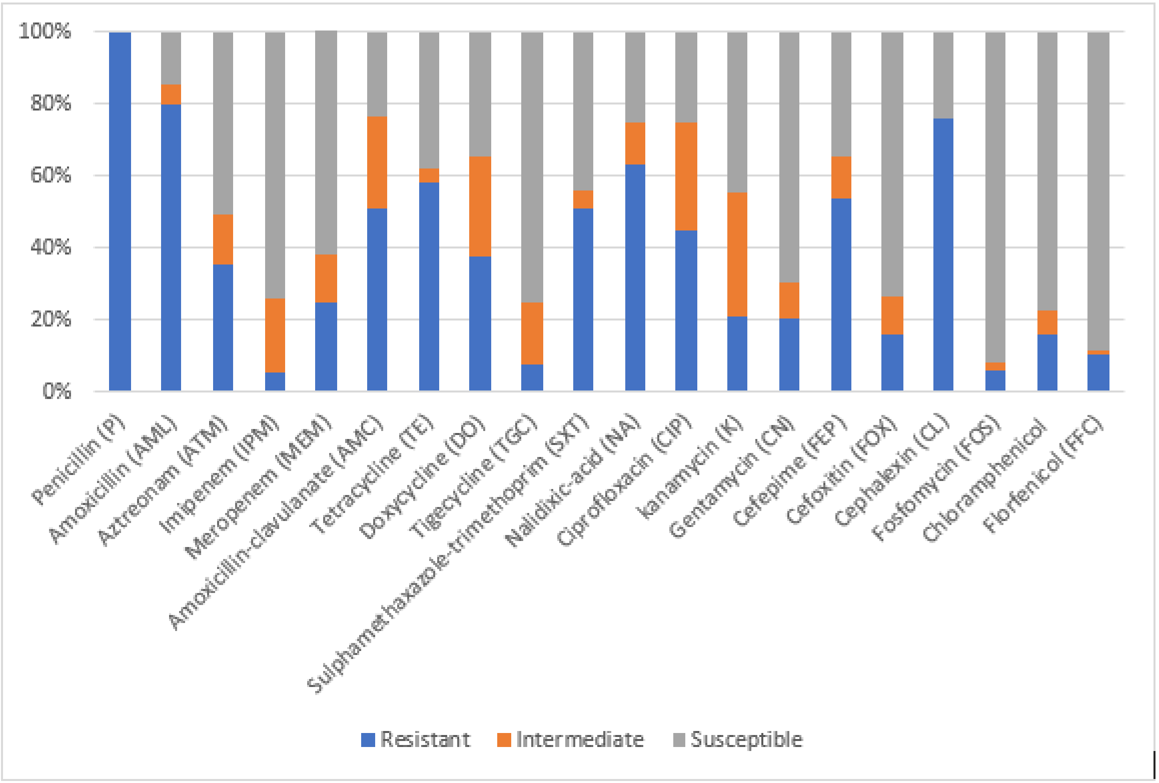

Figure 4| Antibiotic susceptibilities, intermediate, and resistance recorded for 132 *E. coli* isolates by the disk diffusion.

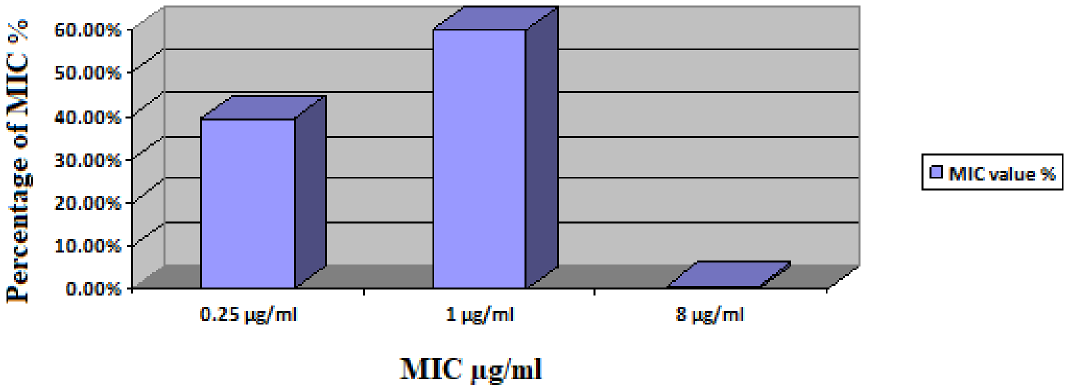

Figure 2 **Minimal inhibitory concentration value for *E. coli* (n=132) isolates.**

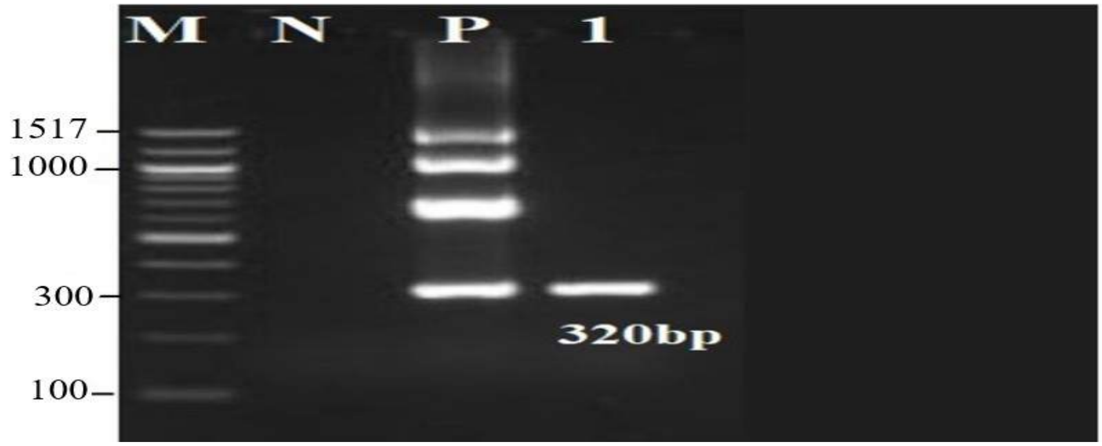

Figure 3 **Electrophoresis for a single *E. coli* isolate carries *MCR*-1gene.** Lane M: 100bp ladder; lane 1: A sample *E. coli* isolate carries *MCR*-1gene; lane P: positive for (*MCR* 1-5) except *MCR* -3, and N: negative controls**.**
